# Biodegradable Polymers: Properties, Applications, and Environmental Impact

**DOI:** 10.3390/polym17141981

**Published:** 2025-07-18

**Authors:** Rashid Dallaev, Nikola Papež, Mohammad M. Allaham, Vladimír Holcman

**Affiliations:** 1 Department of Physics, Faculty of Electrical Engineering and Communication, Brno University of Technology, Technická 2848/8, 61600 Brno, Czech Republic; papez@vut.cz (N.P.); holcman@vut.cz (V.H.); 2 Central European Institute of Technology, Purkyňova 656/123, 61200 Brno, Czech Republic; allaham@vutbr.cz; 3 Institute of Scientific Instruments of Czech Academy of Sciences, 61200 Brno, Czech Republic

**Keywords:** biodegradable polymers, bioplastics, polylactic acid (PLA), polyhydroxyalkanoates (PHAs), renewable feedstocks, starch-based plastics, sustainable packaging, microbial fermentation, environmental biodegradability

## Abstract

The accelerating global demand for sustainable materials has brought biodegradable polymers to the forefront of scientific and industrial innovation. These polymers, capable of decomposing through biological processes into environmentally benign byproducts, are increasingly seen as viable alternatives to conventional plastics in sectors such as packaging, agriculture, and biomedicine. However, despite significant advancements, the field remains fragmented due to the diversity of raw materials, synthesis methods, degradation mechanisms, and application requirements. This review aims to provide a comprehensive synthesis of the current state of biodegradable polymer development, including their classifications, sources (natural, synthetic, and microbially derived), degradation pathways, material properties, and commercial applications. It highlights critical scientific and technological challenges—such as optimizing degradation rates, ensuring mechanical performance, and scaling up production from renewable feedstocks. By consolidating recent research findings and regulatory considerations, this review serves as a crucial reference point for researchers, material scientists, and policymakers. It strives to bridge knowledge gaps in order to accelerate the deployment of biodegradable polymers as integral components of a circular and low-impact material economy.

## 1. Introduction

Biodegradable polymers are defined as materials capable of breaking down and being metabolized by natural microorganisms—such as bacteria, fungi, and algae—ultimately into carbon dioxide and water. The main advantage of these materials is their decomposition under the influence of the environment (biodegradability), and their final products are safe and environmentally friendly. The biodegradability of a polymer material (PM) is influenced more by its molecular structure, chemical bonds, and the presence of substituents than by the source of its raw material. Key factors include molecular weight, chain length, and micro-/macrostructure [1,2,3,4,5,6].

**Figure 1:**
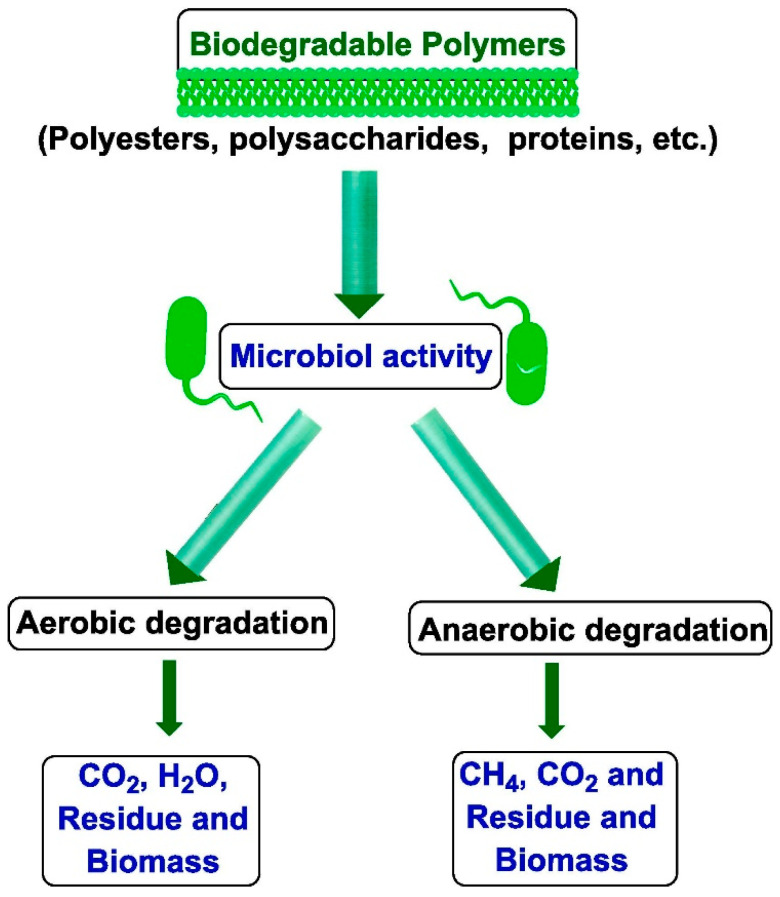
Schematic representation of polymer degradation via biological processes [8]. (Permission to use was granted by Elsevier).

It is important that during degradation, these polymers do not generate any substances harmful to the natural environment. This process differs from composting, which involves creating optimal environmental conditions to accelerate microbial degradation, as illustrated in Figure 1. It is critical to harness nature’s vast bioresources through extensive fundamental research to develop effective, environmentally safe, and economically viable technological methodologies for converting biomass—carbohydrates, proteins, lipids, and terpenoids—into industrially feasible polymeric materials. This opens the door to new material production paths in light of increasing sustainability concerns. Many polymers derived from renewable resources can also be made biodegradable under appropriate conditions [7].

While a wide range of materials are technically biodegradable, substances like plastics and glass may require centuries to fully decompose [9]. Typically, the biodegradation of polymers begins with biodeterioration, a phase in which the material’s physical, chemical, and mechanical integrity is compromised by non-biological (abiotic) environmental factors [8].

A degradable plastic is engineered to experience substantial changes in its chemical structure when exposed to certain environmental conditions, leading to a measurable decline in its physical properties. This transformation is assessed using standardized tests appropriate to the type of plastic and its intended use within a specified timeframe [10]. Several standardization bodies have worked to develop definitions for (bio)degradable plastics. Generally, degradation is understood as a detrimental alteration in a plastic’s chemical structure, physical properties, or appearance.

Biodegradable plastics are used extensively in single-use applications where their environmental decomposition is a benefit, such as in food service, agriculture, and the packaging of perishable goods. A special category comprises biomedical resorbable polymers, used in sutures, wound dressings, screws, plates, and drug delivery agents [11]. The plasticity and elasticity of biodegradable polymers are achieved by incorporating plasticizers such as glycerol [12]. For large-scale manufacturing, polymers must exhibit sufficient thermal stability to avoid degradation during processing while maintaining their molecular weight and properties. Degradation is affected by time, temperature, impurities, and catalyst concentration [13].

While the importance of recycling bio-based, biodegradable plastics—such as bio-polyethylene terephthalate (bioPET), bio-polyethylene (bioPE), and bio-polypropylene (bioPP)—is evident, their end-of-life scenarios are more complex. Biodegradability is often seen as the only acceptable disposal route, yet it does not allow for material or monomer recovery, unlike mechanical or chemical recycling, which preserves resources. As bioplastic production increases, it is critical to determine optimal end-of-life strategies for each major bioplastic [14].

As outlined in the now-withdrawn ASTM D5488 94de1 standard, biodegradable polymers are described as substances that break down into carbon dioxide, methane, water, inorganic elements, or biomass primarily through microbial enzymatic activity, all occurring within a specified period and in controlled disposal environments [15]. The Japan Bioplastics Association (JBPA) defines biodegradability as the capacity of a material to be microbiologically decomposed into carbon dioxide and water, which are reintegrated into natural cycles. This must not be confused with disintegration, which refers merely to the material fragmenting into smaller pieces. Plastics can be certified as “green” only if they meet strict standards, including criteria on heavy metals and safe intermediates.

Certified standards for biodegradable polymers include the following [16]:

- ISO 17088:2021 [ISO 17088:2021; Plastics—Organic recycling—Specifications for compostable plastics. International Organization for Standardization: Geneva, Switzerland, 2021.];
- EN 13432:2000 [EN 13432:2000; Packaging—Requirements for packaging recoverable through composting and biodegradation—Test scheme and evaluation criteria for the final acceptance of packaging. European Committee for Standardization: Brussels, Belgium, 2000.], EN 14995:2006 [EN 14995:2006; Plastics—Evaluation of compostability—Test scheme and specifications. European Committee for Standardization: Brussels, Belgium, 2006.];
- ASTM D6400-12 [ASTM D6400 12; Standard Specification for Labeling of Plastics Designed to be Aerobically Composted in Municipal or Industrial Facilities. ASTM International: West Conshohocken, PA, USA, 2012.].

Biomaterials, per the ASTM, are defined as organic materials with carbon derived from renewable resources via biological processes. ASTM standards for assessing bio-based content via carbon isotope analysis include the following [15]:

- ASTM D6866-12 [ASTM D6866 12; Standard Test Methods for Determining the Biobased Content of Solid, Liquid, and Gaseous Samples Using Radiocarbon Analysis. ASTM International: West Conshohocken, PA, USA, 2012.];
- ASTM D7026-04 [ASTM D7026 04; Standard Guide for Sampling and Reporting of Results for Determination of Biobased Content of Materials via Carbon Isotope Analysis. ASTM International: West Conshohocken, PA, USA, 2004.].

Most biodegradable polyesters are synthesized through the ring-opening polymerization of six- or seven-membered lactones. Among the various classes of biodegradable polymers, aliphatic polyesters are considered leading candidates due to their ability to hydrolyze or enzymatically break down into hydroxycarboxylic acids, which are typically metabolized further [17]. Aliphatic polyesters are among the few high-molecular-weight polymers that are truly biodegradable [18].

Critical properties of the polymer matrix, such as the glass transition temperature (Tg), indirectly affect degradation rates. Although biodegradability is independent of raw material origin, biomass represents a rich, renewable, and carbon-neutral source for biodegradable materials. Nature produces over 200 billion tons of biomass annually via photosynthesis, of which 75% falls into the carbohydrate class; yet, only 3.5% is utilized by humans [19,20,21,22]. Several parameters influence the degradation behavior of biodegradable polymers, with the most significant being chemical composition, molecular weight and its distribution, crystallinity, and (micro)structure. Recent research also indicates a strong impact of monomer sequence regularity on degradation properties [23]. The development of biodegradable fibers derived from aliphatic polyesters has been widely explored for their use in medical applications [24,25,26,27].

### 1.1. Current Trends and Challenges in the Field of Biodegradable Polymers

One of the major challenges faced by researchers all around the globe is the development of technological solutions aimed at creating synthetic polymers with accelerated biodegradability [28]. Bioplastics can be either bio-based (i.e., derived from renewable resources) or biodegradable (i.e., capable of decomposing into natural elements) [29]. One promising approach is the creation of synthetic additives or modifiers that can actively regulate the rate of biodegradation and significantly accelerate the degradation process of major industrial polymers such as polyolefins, polystyrene, and phthalates [30].

The lack of biodegradability in other polymers may impact their long-term environmental performance compared to biodegradable systems [31]. In nature, these polymers undergo significant transformations, including hydrolysis by water and oxidation by atmospheric oxygen, which alter their physical characteristics. Furthermore, such polymers can serve as substrates for the growth of specific microorganisms [32].

A biofilm is a community of microbial cells associated with a surface and embedded in a matrix of extracellular polymeric substances, consisting of 80–95% water and hydrated biopolymers (mainly polysaccharides) [33]. According to Donlan and others [33,34], biofilm formation causes the micro-swelling of polymer surfaces, making them more vulnerable to microbial attack.

Currently, researchers are focused on three major areas [35]:

- Introducing functional groups into biodegradable polymers to promote photodegradation;
- Creating composites of conventional polymers with natural biodegradable additives that initiate breakdown;
- Synthesizing new biodegradable plastics using existing synthetic industrial products.

Several technologies have been developed to impart biodegradability to traditional polymers [28,36]. These include the following:

1. Introducing agro-industrial waste products (e.g., beet pulp, oat husks, buckwheat hulls, corn mash) as additives into synthetic polymers.
2. Creating composite materials based on synthetic and natural biodegradable polymers (e.g., starch, cellulose, polylactic acid).
3. Adding oxo-biodegradable additives to synthetic polymers, which contain transition metal salts that generate free radicals, leading to hydroperoxide and peroxide formation, which promotes biodegradation [37,38].

The use of hydrogen in the production of PGA is well established [39]. This process transforms organic substances, including waste, into bioplastics by initially gasifying them into carbon monoxide and hydrogen. These gases are then assimilated by photosynthetic bacteria into the cellular biomass under oxygen-free (anaerobic) conditions [40]. Acidogenic fermentation can also produce a sufficient hydrogen yield [41], which can be used for bioplastic production [42,43].

New biodegradable biopolymers are being developed using biotechnological processes. These are referred to as “green plastics” and are derived from plants. Green plastic is of significant interest to modern researchers as a sustainable alternative to traditional petroleum-based plastics. It must originate from renewable sources, be inherently biodegradable, and be environmentally friendly [44].

The production of low-cost raw bioplastics is feasible through the use of mixed microbial cultures under non-sterile cultivation conditions. The organic acids generated during the acidogenic fermentation of municipal solid waste (MSW) can serve as a predominant carbon source for the biosynthesis of raw bioplastics. The environmental benefits of producing and utilizing raw bioplastics derived from the organic fraction of MSW include the following:

- Reducing the volume of waste destined for incineration;
- Lowering the amount of ash requiring landfill disposal;
- Enabling the use of seawater for MSW separation, thereby conserving freshwater resources.

In 2019, bio-based polyamides accounted for approximately 12% of the global bioplastics market. Commercially available bio-based polyamides are typically derived from sebacic acid or undecanoic acid, both of which can be sourced from castor oil. Among these, polyamide 11 (PA11) is the most common and commercially available. However, other polyamides, such as PA610, PA1010, PA510, PA6, PA66, and PA12, can also be produced in fully or partially bio-based versions [45,46].

Products requiring rapid photodegradation primarily include packaging materials such as shopping bags, garbage bags, snack wrappers, and wrapping films for paper goods. Additionally, disposable tableware, drinking cups, egg cartons, dairy cartons, and personal hygiene products (e.g., diapers, tampon applicators, and bandages) are often cited as candidates for photodegradable materials. Notably, degradability in these cases is only relevant if such products are not properly disposed of; otherwise, photodegradability serves mainly to reduce littering impact [47]. The development of photo- and biodegradable plastics relies on the incorporation of photo- and bioactivating additives into the polymer chain, which should contain functional groups capable of degradation under ultraviolet radiation or anaerobic bacterial activity [35].

Raw bioplastics containing polyhydroxyalkanoates (PHAs) can be applied across multiple industries, particularly in construction and agriculture [48]. However, there are two potential challenges associated with the use of raw PHA-based nanocomposite bioplastics. The first is the high melting temperature of PHAs, typically in the range of 160–180 °C [49,50].

The melting temperature of PHB (polyhydroxybutyrate) is close to its thermal degradation point (Td) [51]. Similarly, the degradation temperatures of proteins, polysaccharides, and polynucleotides are also in this range, indicating that all biopolymers exhibit poor thermal stability near the melting point of PHAs. Natural antioxidants found in biomass can help reduce the rate of thermal degradation of biopolymers [52]. Protein itself can be regarded as a thermoplastic material when combined with plasticizers that suppress crosslinking reactions, which might otherwise result in the formation of thermosetting materials [53]. Therefore, the molding process for composite raw PHA-containing bioplastics should be kept as brief as possible to minimize the thermal degradation of PHAs and other bacterial biopolymers [54,55].

Currently, four main scientific approaches have been developed for the production of these environmentally friendly and sustainable bioplastics: (1) the partial modification of natural polymers (starch, cellulose, pullulan); (2) monomer production using de novo or fermentation processes followed by traditional chemical polymerization (e.g., PLA, polyethylene); (3) microbial cultivation and adaptation, including the use of genetically engineered colonies (e.g., PHA, PHB) [56]; and (4) the production of partially biodegradable polymers such as polybutylene terephthalate (PBT), poly(butylene adipate-co-terephthalate) (PBAT), polybutylene succinate (PBS), and polyurethane (PU) [57]. However, most commercially available biodegradable bioplastics degrade slowly under environmental conditions, even in the presence of microorganisms, as they are often designed to degrade in specific environments such as industrial composting facilities [58].

### 1.2. Relevance

The global urgency to reduce plastic pollution and reliance on fossil-based polymers has intensified the need for sustainable alternatives. Biodegradable polymers have emerged as promising materials capable of mitigating environmental degradation while supporting a circular economy. Their relevance spans across sectors such as packaging, agriculture, medicine, and consumer goods, where single-use plastics dominate. The increasing volume of plastic waste—projected to exceed 1.1 billion tons by 2050—has catalyzed a shift in policy, industry practices, and research priorities, all pointing towards biodegradable solutions. This review of biodegradable polymers is timely and essential, considering the current limitations of both industrial-scale adoption and the public understanding of these materials. This comprehensive synthesis of biodegradable polymer classifications, degradation pathways, and applications will enable stakeholders—from researchers to regulators—to make informed decisions that align with ecological and economic sustainability goals. Moreover, this review underscores the multidisciplinary nature of biodegradable polymer development, incorporating advances in microbiology, chemistry, materials science, and environmental engineering. As the push for zero-waste manufacturing intensifies, biodegradable polymers represent a pivotal innovation in reconciling industrial productivity with environmental stewardship.

### 1.3. Methodology

This review adopts a comprehensive literature-based methodology aimed at consolidating and synthesizing contemporary research on biodegradable polymers. Scientific publications, technical reports, and regulatory standards were systematically analyzed to map the current landscape of biodegradable materials. Sources include peer-reviewed journals in polymer science, materials engineering, biotechnology, and environmental science, with a particular focus on recent advances post-2010. The methodology encompasses three key analytical dimensions: (1) the classification and synthesis of biodegradable polymers, distinguishing natural, synthetic, and microbially derived materials; (2) an evaluation of degradation mechanisms—both abiotic and biotic—and their environmental dependencies; and (3) a critical assessment of application-specific challenges and commercial viability across industries. Special emphasis is placed on polylactic acid (PLA) and polyhydroxyalkanoates (PHAs) given their dominance in current research and market penetration. Additionally, the review incorporates data from relevant standards organizations (e.g., ASTM, ISO, JBPA) to contextualize biodegradability within regulatory frameworks. This methodological approach ensures a balanced representation of scientific innovation, practical deployment, and policy implications, enabling a holistic view of the biodegradable polymer domain.

## 2. Classifications of Biopolymers and Biodegradation Mechanisms

### 2.1. Classifications of Biopolymers

Over the past two decades, there has been increased interest in polymers derived from renewable resources due to limited petroleum reserves and environmental concerns, such as waste accumulation and a resistance to degradation. The development of new biodegradable polymers based on plant-derived biopolymers and their derivatives, in combination with synthetic polymers, offers opportunities for innovative degradable systems [59,60,61]. The majority of researched biodegradable polymers are part of the polyester group, with a particular emphasis on poly(glycolic acid), poly(lactic acid), and their copolymers. These remain crucial in medical applications requiring resorbable materials [62]. To qualify as biodegradable, materials must pass a series of tests, including chemical composition (e.g., heavy metals), complete degradation under laboratory and real-world conditions, and the ecotoxicity of the resulting compost. Biodegradation should reduce plastic fragments to sizes below 2 mm in over 90% of the sample under real conditions [63].

Photodegradable polymers include ethylene–carbon monoxide copolymers. Vinyl ketone monomers serve as photo-initiators for the degradation of base polymers such as PE (polyethylene) and PS (polystyrene). When introduced in amounts of 2–5% as comonomers, these materials retain properties similar to PE or PS but become susceptible to photodegradation under UV light within the range of 290–320 nm [64]. Even a small number of keto groups in the polymer backbone renders polyethylene (PE) photodegradable, a desirable characteristic given current environmental pollution issues [65].

For many plastic types, hydrolytic degradation is the most common environmental decomposition route [66]. A powerful alternative is photodegradation. Intrachain keto groups are particularly effective for promoting photodegradation as they enable chain scission via Norrish Type I and Type II reactions [67].

Based on their raw material, biodegradable polymers can be divided into three categories [68]:

- Edible or cellulose-based packaging from biomass of terrestrial or marine origin (proteins, fats, polysaccharides);
- Polyesters synthesized from renewable and petroleum-based sources with properties similar to conventional plastics;
- Polyhydroxyalkanoates (PHAs) and similar biopolymers obtained from microbial fermentation.

Bioplastics are generally classified into three main categories [69]:

- Conventional plastics derived from fossil resources but modified to be biodegradable, such as PBAT;
- Non-biodegradable or partially biodegradable plastics, including bio-based polyethylene (PE), polypropylene (PP), polyethylene terephthalate (PET), and technically advanced biodegradable plastics such as polytrimethylene terephthalate (PTT) and thermoplastic polyester elastomers;
- Plastics that are both bio-based and biodegradable, for example, PLA (polylactic acid) and PHAs (polyhydroxyalkanoates).

### 2.2. Degradation Types and Mechanisms

The factors that influence the mechanical properties of biodegradable polymers are well known to polymer scientists. Key variables influencing polymer properties include the selection of monomers, the type of initiator employed, processing parameters, and the incorporation of additives. These factors collectively determine characteristics such as hydrophilicity, the degree of crystallinity, melting point, glass transition temperature, molecular weight and its distribution, the nature of end groups, the arrangement of monomer sequences (whether random or block), and the residual presence of unreacted monomers or added substances. The biodegradation of polymer composites is a two-stage process, consisting of abiotic and biotic oxidation. During the mineralization of plastics, low-molecular-weight polymer residues undergo enzymatic dissimilation—breaking down with the release of energy. This dissimilation process is intrinsically linked with assimilation. The assimilation of plastics refers to the metabolic processes by which microorganisms incorporate plastic components into their cellular metabolism, resulting not only in energy release but also in biomass growth and the formation of secondary metabolites. These assimilation features provide irrefutable evidence of the ongoing biodegradation of the polymer [70,71]. A novel biodegradable polyester was synthesized via the chain-extension of poly(p-dioxanone) (PPDO) with poly(butylene succinate) (PBS) [72].

Numerous studies and patents have explored the enzymatic breakdown of biodegradable aliphatic polyesters—such as PLA, PBS, PCL, PTT, and poly(butylene adipate) (PBA)—typically utilizing enzymes like lipases or proteinase K. These processes often continue with the further degradation of the produced oligomers. Despite this progress, enzymatic depolymerization remains a developing technology. Its current limitations include a relatively slow reaction rate, especially for polymers with a high crystallinity and strong intermolecular interactions. Moreover, enhancing the reaction speed by raising the temperature is not feasible, as it risks deactivating the enzymes involved [73]. The field of biodegradable polymeric materials has made significant progress in recent years.

There are five main types of synthetic polymer degradation, classified by external factors [74]:

1. Bacterial degradation;
2. Chemical degradation;
3. Photodegradation due to sunlight;
4. Thermal degradation;
5. Mechanical degradation.

Polymer biodegradation occurs via two primary mechanisms: biological hydrolysis and biological oxidation [75,76]. Hydrolysis is mediated by specific depolymerase enzymes, while oxidation can occur non-enzymatically. Both these destructive processes act synergistically [77].

To initiate biodegradation, a composite should contain the following [78]:

1. Heteroatoms;
2. Biodegradable bonds (R = CH_2_; R = CH–R1; R–CH_2_–OH; R–CH(OH)–R; R–CO–H; R–CO–R1, etc.);
3. Carbon chain fragments with fewer than five CH_2_ groups;
4. Bulky substituents;
5. Natural fillers that support microbial metabolism: starch, cellulose, lactose, magnesium, and urea.

Depending on the degradation mechanism, biodegradable polymers can be categorized into three main groups [79,80]:

1. Biodegradable polymers—natural polymers such as cellulose, starch, agro-industrial waste, beet pulp, natural rubber, polyhydroxybutyrate (PHB), polybutyrolactone, polylactic acid (PLA), etc.;
2. Polymers subject to biodeterioration—such as aliphatic polyesters and polyamides;
3. Materials susceptible to bioerosion—typically blends or copolymers of synthetic polymers with natural polymers from Group 1 (e.g., polyethylene with starch).

### 2.3. Types of Biomaterials for Creating Biodegradable Coatings

Biodegradable polymers often used for coatings can be broadly categorized into natural, semi-synthetic, and synthetic types based on their origin and method of production. Each category encompasses materials with unique physicochemical properties and specific applications, particularly in agriculture, biomedicine, and environmental sustainability. The following classification highlights representative examples of biodegradable polymers along with their defining characteristics and practical uses.

1. Natural Polymers:
  Several notable examples of natural polymers include the following:
  - Starch is biodegradable, thus reducing environmental pollution. Starch-based coatings can slow the release of fertilizers, allowing plants to absorb nutrients gradually, minimizing leaching into groundwater and reducing application frequency. Starch films also exhibit good mechanical strength and flexibility, making them suitable for encapsulation purposes [81].
  - Gelatin is biocompatible and biodegradable. Due to its high water content, gelatin has low mechanical strength. To enhance its elasticity, additives such as other polymers or organic/inorganic compounds are commonly used [82].
  - Wheat gluten can be processed to produce bioplastics [83,84].
  - Collagen supports structural processes, cell growth, proliferation, and migration. It is biocompatible, biodegradable in tissue environments, and non-cytotoxic, making it an ideal material for rapid tissue scaffold formation [85].
2. Chitosan: Chitosan protects plants from pathogens due to its antibacterial properties, improving plant health. Chitosan is considered semi-synthetic; it is a naturally occurring biopolymer obtained by chemically modifying chitin, a structural polysaccharide present in the exoskeletons of marine crustaceans, certain insects, and the cell walls of fungi. This transformation is typically achieved through a deacetylation process, wherein acetyl groups are removed from chitin to yield chitosan, which imparts distinct physicochemical and biological properties [86,87,88]. It is highly biocompatible and safe for agricultural use, and it enables the controlled release of fertilizers [89]. Chitosan is biodegradable, non-toxic, and exhibits antimicrobial properties [90,91,92].
3. Synthetic Polymers:
  Below are a couple of notable examples of synthetic polymers:
  - Polyethylene glycol (PEG) has tunable permeability based on temperature and humidity, enabling controlled nutrient release. Due to its hydrophilicity, PEG coatings help retain soil moisture and enhance plant nutrient uptake [93].
  - Lactate-based polymers, derived from lactic acid, degrade rapidly in nature and break down into harmless byproducts like CO_2_ and water. These coatings can shield fertilizers from harsh environmental conditions until they are needed by plants [94].

Composite films made from a combination of chitosan, microcrystalline cellulose fibers, and gelatin have enhanced strength and biodegradability when buried in soil, making them suitable for packaging and tray manufacturing [95]. A growing method for imparting biodegradability to synthetic polymers involves blending them with natural biodegradable polymers like cellulose, starch, chitin, and chitosan. This approach began with materials filled with carbohydrate-based polymers, particularly starches [96,97].

## 3. Prominent Biodegradable Polymers

### 3.1. Polylactic Acid or Polylactide (PLA)

Among the most promising biodegradable plastics for packaging is polylactic acid (PLA)—a condensation product of lactic acid. The industrial synthesis of PLA occurs via the ring-opening polymerization of lactide [98,99,100] or via the azeotropic polycondensation of lactic acid [99]. Despite its advantages, PLA has a relatively slow degradation rate (half-life of approximately 168 days). Therefore, copolymerization with monomers like glycolide—derived from glycolic or monochloroacetic acid—is being explored to tailor its biodegradation profile [101]. PLA is a biodegradable thermoplastic widely recognized for its favorable mechanical strength, biocompatibility, and non-toxic degradation products, making it suitable for diverse applications ranging from packaging to biomedical devices [102]. Its potential in drug delivery has been demonstrated through numerous studies involving PLA and its copolymers [103,104,105,106,107]. Over the past decade, substantial advances have been made in the controlled polymerization of synthetic PLA. Among the various synthesis techniques explored [108,109], the ring-opening polymerization (ROP) of lactide has emerged as the most effective method [110].

PLA was first synthesized in 1932 by DuPont scientist Wallace Carothers and became the first industrially produced bio-based and biodegradable polymer in the late 1990s [111]. It remains one of the most commercially viable biodegradable polyesters due to its biocompatibility and biodegradability. One of the reasons for this is that lactic acid is easily produced via a biotechnological process (usually based on a strain of lactobacilli) from inexpensive raw materials [112].

PLA is recognized for its dual functionality: it is biodegradable, making it suitable for short-term uses such as packaging, and biocompatible, allowing for its safe application in medical contexts like implants, sutures, and drug delivery systems. PLA undergoes abiotic degradation through the hydrolysis of its ester bonds, which does not require enzymatic assistance. In the subsequent phase of its breakdown, enzymes further degrade the resulting oligomers into simpler compounds, eventually leading to complete mineralization via biotic processes. As its primary building block—lactic acid—is derived from renewable carbohydrate sources through fermentation, PLA aligns well with global sustainability goals and is widely regarded as an eco-friendly material [113].

Polylactic acid (PLA) undergoes degradation in aqueous environments through the hydrolysis of its ester linkages. Similar to polycaprolactone (PCL) and poly(propylene carbonate) (PPC), PLA degrades slowly under neutral pH conditions but exhibits significantly accelerated degradation in alkaline environments compared to acidic ones [114].

**Figure 2:**
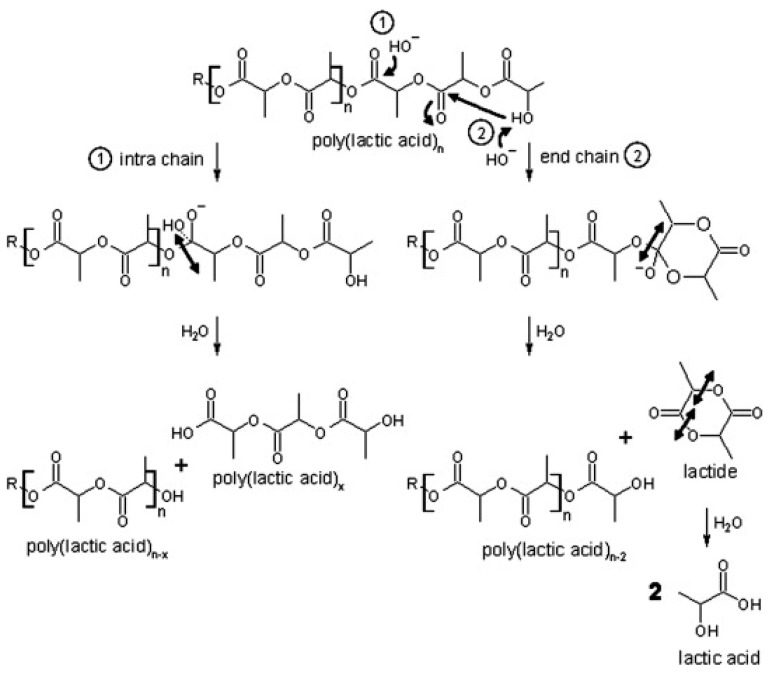
Hydrolytic degradation of PLA under basic conditions [115]. (Permission to use was granted by Elsevier).

PLA depolymerization in basic conditions occurs via the gradual release of dimer units (see Figure 2). This process likely involves intramolecular transesterification at the polymer chain end. Under basic catalysis, the hydroxyl end-group performs an electrophilic attack on a neighboring carbonyl group, leading to ring formation. This step results in polymer chain shortening as the newly formed lactide is hydrolyzed. Subsequently, the lactide is further broken down into two lactic acid molecules. Additionally, random base-catalyzed attacks on the ester groups in the polymer backbone initiate intramolecular degradation, producing low-molecular-weight compounds through ester bond cleavage [115].

Lactic acid can be converted into its dehydrated dimer, lactide, which is then polymerized through ring-opening polymerization to produce high-molecular-weight polymers. These polymers can also be copolymerized with caprolactone to create valuable packaging films. Additionally, the bacterial fermentation of substrates such as glucose and acetic acid produces novel thermoplastic polyesters like poly(3-hydroxybutyrate) (PHB) [116]. Among biopolymers, those derived from crystalline nanocellulose sourced from agricultural materials exhibit the fastest biodegradation rates. The presence of E. faecium was found to cause the greatest decrease in degradation rate, while also slightly enhancing tensile strength when compared to strains of *P. acidilactici* [117].

One of the main limitations of polylactic acid (PLA) lies in its relatively low glass transition temperature (Tg) of 55–60 °C, above which the material becomes tacky. This characteristic, combined with its slow crystallization kinetics, complicates the drying and crystallization of amorphous PLA waste, such as films. Similarly, thermoplastic starch suffers from hydrolytic degradation during use, restricting the recyclability of such materials to lower-value applications. In addition, thermoplastic starch is immiscible with conventional packaging plastics and cannot be effectively co-processed into high-performance secondary products [118].

An increasing volume of research underscores PLA’s status as one of the most promising biodegradable polymers [119]. It is processable by standard industrial techniques—including injection molding, blow molding, thermoforming, and extrusion—and is commercially available in a variety of grades. Its biodegradability supports short-lived applications such as in packaging, while its compatibility with biological systems makes it ideal for medical uses, including sutures, implants, and drug encapsulation.

PLA is generally synthesized using homoleptic metal-based catalysts (e.g., tin, aluminum, and zinc). However, achieving high stereocontrol and catalyst activity has proven challenging due to complex equilibria and the formation of multinuclear species. Research into alternative catalysts, such as homoleptic yttrium alkoxides, continues to expand [120,121].

The degradation of PLA occurs abiotically via the hydrolysis of ester bonds, a process that does not require enzymatic catalysis. Biodegradation proceeds in two stages: initial abiotic hydrolysis, followed by microbial enzymatic activity that converts oligomers into mineralized end products. Given that PLA’s primary monomer—lactic acid—can be sourced from the fermentation of renewable carbohydrates, the polymer aligns with global sustainability initiatives and is regarded as an eco-friendly alternative to petroleum-based plastics [122]. In terms of processability, PLA is compatible with conventional plastic manufacturing methods and is increasingly being used in applications such as molded articles, fibers, textiles, and food packaging [123,124,125,126].

Despite its advantages, PLA remains more costly than traditional polymers like polyethylene and polystyrene. However, recent efforts aim to reduce its production costs through less energy-intensive manufacturing. To improve polymerization stereoselectivity, novel catalysts based on zirconium, hafnium, gold, and platinum are being explored [100]. Comprehensive overviews of PLA synthesis and industrial relevance are available in recent reviews [127,128].

PLA can be tailored to exhibit a wide range of chemical and mechanical properties depending on the synthesis method. This flexibility in synthesis allows PLA and its copolymers to be customized for high-performance applications, particularly in tissue engineering, where their biocompatibility and mechanical integrity support tissue regeneration [129].

The monomer lactic acid, a naturally occurring organic acid, is typically produced via the microbial fermentation of renewable feedstocks such as sugarcane. As a result, PLA is considered environmentally friendly and suitable for use in biomedical contexts due to its non-toxicity and renewable origin. The authors of [130] report on the fermentation-based production of lactic acid and subsequent polymerization routes for PLA, with an emphasis on its biomedical applications and relevance in sustainable material development.

Poly(L-lactic acid) (PLLA), a semi-crystalline form of PLA derived from L-lactide, typically exhibits a tensile modulus of ~3 GPa and tensile strength around 60 MPa, along with high transparency and good processability [99]. Its crystallinity (~37%) contributes to its wide usage in packaging, including disposable cups, containers, films, and bottles. PLA fibers, produced via thermal spinning, exhibit properties comparable to those of PET and nylon [131,132,133]. These fibers can be fabricated by either solvent-based or melt-spinning techniques, with solvent-spun fibers often offering superior mechanical properties due to reduced thermal degradation during processing [134].

Beyond its packaging and biomedical uses, PLA is employed in the production of textiles, hygienic products, disposable tableware, agricultural mulch films, and even in a foamed form as a sustainable alternative to polystyrene for insulation and cushioning applications [135,136,137].

For convenience, the key points are summarized and provided in Table 1.

### 3.2. Polyhydroxyalkanoates (PHAs)

PHAs are bio-derived and biodegradable aliphatic polyesters synthesized through the polymerization of β-, γ-, and δ-hydroxyalkanoic acids. These acids are primarily obtained via the fermentation of sugars and lipids (e.g., glucose, sucrose, and vegetable oils) derived from a broad range of raw materials [15,138]. This exciting class of polyesters offers biodegradability, thermoplasticity, and favorable mechanical properties. They can also be produced by various microbial strains using renewable resources under stress conditions—such as carbon excess and limitations in nitrogen, oxygen, or phosphorus [139].

Similar to PLA, PHAs are used in various disposable applications across the packaging and biomedical sectors. Owing to their excellent biocompatibility, specific types like PHB and PHBV are currently under investigation for biomedical uses such as bioresorbable surgical sutures, wound healing materials, tissue engineering scaffolds, bone fixation devices, and porous membranes that support soft tissue regeneration [16,140]. Biopolymers based on cellulose, starch, PHAs, bio-derived polyethylene, and PLA are also employed in agricultural applications, including the production of shading nets and biodegradable mulching films [141,142,143]. Additionally, PHAs have been utilized in the formulation of printing toners and as components in coating adhesives [144]. Their potential in agriculture further extends to applications such as seed coatings, encapsulated slow-release fertilizers, biodegradable films for crop protection, and compostable containers for greenhouse use [145].

**Figure 3:**
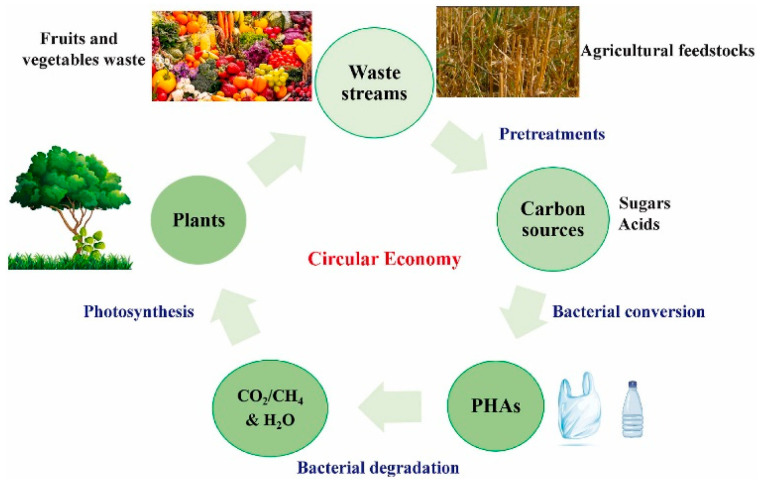
Schematic representation of a closed-loop system for producing PHAs from waste materials [146]. (This Figure is available as open access).

Figure 3 illustrates the overall closed-loop process of producing polyhydroxyalkanoates (PHAs) using waste-derived feedstocks. Typically, complex organic materials present in waste streams are first broken down into simpler sugars, and are then fermented—often under anaerobic conditions—into volatile fatty acids (VFAs). While pretreatment methods can aid this conversion, they often raise processing costs and may produce toxic byproducts such as furfural, which can hinder PHA biosynthesis. To ensure the reliable and sustainable generation of sugars and VFAs from waste, it is essential to fine-tune operational parameters. Moreover, controlling or preventing acidogenic inhibition during fermentation is crucial. Therefore, implementing environmentally friendly, efficient, and economically viable pretreatment strategies is key for large-scale applications [146].

The properties of PHAs vary with the length and structure of the side chains in their repeating units, and the type of polyester formed depends on the microbial strain used. PHAs accumulate as intracellular granules (0.2–0.7 µm in diameter) in bacterial cytoplasm [147]. Their property profiles can be tuned through substrate selection, bacterial strain, and fermentation conditions.

Plant biomass can serve as a feedstock, contributing to a closed carbon cycle [148]. Sustainable carbon sources include biomass, municipal waste, and industrial waste streams, which reduce environmental waste and production costs since 30–50% of total PHA production costs arise from raw materials [149]. Other sustainable inputs include wood chips, cardboard scraps, and waste from plastic bottles and bags [150,151].

Naturally occurring biopolymers serve as biological storage systems or protective mechanisms. Microalgae play a crucial role in biological carbon fixation through photosynthesis, ultimately leading to the synthesis of branched polysaccharides. PHA is a leading microbial-derived biopolymer. For example, rice bran is a viable substrate for biopolymer synthesis catalyzed by a bacterium known as Sinorhizobium meliloti MTCC 100, this method is preferred over other synthetic methods due to its environmental safety and low agro-waste output [152].

Key attributes of PHAs include biodegradability, closed-loop carbon cycling, production from renewable sources, environmental friendliness, low energy requirements, a lack of toxic byproducts, and minimal greenhouse gas emissions. The global PHA market was projected to reach 23,734.65 metric tons by 2021, with a compound annual growth rate of 6.27% [153]. Potential markets for raw PHA-based bioplastics include packaging, food service products, consumer electronics, medical devices, agriculture (biodegradable mulch films), toys, and textiles [154].

The wide range of physical properties of PHA families, as well as the enhanced performance achievable through chemical modification [155] or blending [156,157,158,159], provide them with a broad spectrum of potential applications. These primarily focus on packaging, including containers and films, as well as biodegradable personal care items such as diapers and their packaging [160].

Sustainable PHA production must consider four “E” aspects: economic, ethical, environmental, and engineering [161]. PHA production can contribute to a reduction in greenhouse gas (GHG) emissions (by approximately 200%), decreasing fossil energy consumption (by around 95%), minimizing waste, and supporting bioeconomy concepts [162].

Commercial applications of PHAs include a wide range of packaging uses for everyday items such as razors, shampoo bottles (e.g., by Wella AG), feminine hygiene products, plastic bags, surgical garments, carpets, and upholstery (developed by Biomers, P&G, Metabolix, and other companies) [163].

For convenience, the key points are summarized and provided in Table 2.

### 3.3. Other Noteworthy Biodegradable Polymers

Poly(butylene succinate) (PBS) is used in various applications such as food packaging films, shopping bags, agricultural mulch films, plant pots, and hygiene products. However, its use in the biomedical field is limited due to its low biocompatibility and biological activity. PBS is also employed in blends and composites where fillers are added to enhance thermal conductivity, mechanical strength, gas barrier properties, and flame retardancy [164,165,166].

Polymers synthesized from poly(butylene adipate-co-terephthalate), poly(butylene succinate/adipate), and poly(ε-caprolactone) are considered biodegradable due to the vulnerability of their carbon backbones to breakdown by enzymatic activity [167].

Polycaprolactone (PCL) is notable for its biocompatibility and slow degradation rate in vivo (1–2 years), which makes it suitable for medical applications requiring gradual bioresorption, such as some suture materials, drug delivery systems, and tissue engineering scaffolds [168,169,170]. PCL is synthesized through the ring-opening polymerization of caprolactone monomers, a process closely linked to its degradation behavior [171].

Like other petroleum-derived biodegradable plastics, PCL is also blended with bio-based biodegradable plastics such as starch-based polymers, PLA, PHA, and PBS [172].

Polyvinyl alcohol (PVA) is commonly used in multilayer assemblies for food packaging due to its excellent film-forming ability and oxygen barrier properties. PVA is a biodegradable synthetic polymer known for its excellent film formation, strong adhesion, and high thermal stability. It has become widely used in the materials industry [173,174,175]. Additional applications include water treatment chemicals, dyes, detergents, disinfectants, and agricultural products [176,177,178]. PVA is also widely used in fiber production using various spinning methods, including electrospinning. Its physical properties—such as electrical resistance, water solubility, thermal behavior, and gas permeability—are influenced by its degree of crystallinity, which is determined by the degree of hydrolysis and molecular weight. Crystallinity is also affected by plasticizer content, bound water molecules, and similar factors. Since PVA is a relatively expensive polymer, its blends with cheaper fillers such as starch and cellulose are extensively studied to reduce costs and potentially improve biodegradability [179]. PVA is widely utilized due to its water solubility and ease of biodegradation by microorganisms and enzymes. In biomedical applications, poly(alkyl cyanoacrylates) are commonly used due to their rapid degradation—ranging from hours to days [180,181]. PVA fibers are particularly employed in biomedical fields and to enhance the mechanical properties of binding materials [182,183].

Poly(butylene adipate terephthalate) (PBAT) is widely used in compostable bags for organic waste, agricultural mulch films, packaging wraps, and disposable tableware. It is a biodegradable aliphatic–aromatic random copolyester synthesized via the polycondensation of adipic acid, terephthalic acid, and 1,4-butanediol. PBAT offers excellent flexibility, high elongation at break (up to 700%), good resistance to oil and water, and moderate tensile strength (~30 MPa) [184,185].

Other emerging bio-based polymers include polyethylene furanoate (PEF) and other furan dicarboxylate-based polyesters [186].

Polyglycolide (PGA), also known as polyglycolic acid, is the simplest linear aliphatic polyester. It is a petroleum-derived biopolymer characterized by a straightforward polyester molecular structure [187,188]. Like PLA, it belongs to the group of poly(α-hydroxy acids) and undergoes degradation primarily through hydrolytic bulk erosion in aqueous conditions. This degradation process initiates with a reduction in molecular weight, followed by material mass loss. The rate at which these polymers degrade is largely influenced by their initial molecular weight and the specific ratio used in copolymer formation.

Poly(L-lactide) (PLLA), a semi-crystalline variant of PLA, degrades more slowly and possesses superior mechanical properties, making it particularly well suited for structural or load-bearing applications [189]. Some reports describe the synthesis of PLA with molecular weights reaching 102,000 and exceptionally high melting temperatures (ranging from 210 to 218 °C), attributed to the formation of a distinctive supramolecular structure [100,190,191]. PLLA is recognized for being both biodegradable and biocompatible, offering strong mechanical performance along with favorable chemical and physical stability and low biological toxicity [192,193]. Another important copolymer, poly(lactic-co-glycolic acid) (PLGA), is synthesized via the ring-opening polymerization of lactide and glycolide and is widely known for its biodegradability and compatibility with biological systems [194,195]. Meanwhile, poly(DL-lactide) (PDLLA) is an amorphous polymer, offering different properties from its semi-crystalline counterpart [196]. Poly(trimethylene carbonate) (PTMC), part of the polycarbonate family, is produced through the ring-opening polymerization of trimethylene carbonate using diethylzinc as a catalyst, and copolymers incorporating glycolide and dioxanone have also been developed to enhance its properties [197].

Polyurethanes (PUs) are unique polymeric materials that exhibit a wide range of physical and chemical properties. This versatility has enabled their widespread adoption in modern technologies for applications such as coatings, adhesives, fibers, foams, and thermoplastic elastomers [198]. The biodegradability of polyurethanes is largely dependent on the chemical nature of their segments. By selecting appropriate soft segments, the degradation behavior of the polymer can be tailored. Polyurethanes based on polyester polyols are generally more susceptible to biodegradation, whereas those based on polyether polyols tend to be more resistant [199,200]. Poly(ester urethanes) have been synthesized through the reaction of lysine diisocyanate with polyester diols derived from lactide or ε-caprolactone [201,202]. A novel waterborne polyurethane was synthesized using a rapeseed oil-based polyol as a soft segment. These water-dispersible polyurethanes were employed to modify plasticized starch, aiming to produce new biodegradable materials with enhanced performance [203,204].

Bio-polypropylene (Bio-PP) is derived from renewable resources. Propylene, the second most important monomer for polyolefins after ethylene, is used to produce polypropylene, which held a 20% market share in 2019 [205]. According to Bioplastics Europe, the PP production capacity is expected to increase almost sixfold by 2024 [206].

A critical issue is imparting biodegradability to established industrial polymers such as polyethylene (PE), polypropylene (PP), PVC, polystyrene (PS), and polyethylene terephthalate (PET), which can persist in landfills indefinitely [207].

Polypropylene carbonate (PPC) is synthesized via the copolymerization of propylene oxide and carbon dioxide. It has favorable properties such as compatibility and impact resistance, although its thermal resistance and biodegradability require improvement, typically achieved through blending with other polymers [208].

Polyethylene terephthalate (PET), a type of polyester, has recently been suggested as a biodegradable option for packaging applications [209]. While PET can be recycled, incinerated, or disposed of in landfills, its primary intended end-of-life process is composting, where it undergoes soil degradation to break down into carbon dioxide and water [210].

Polydioxanone (PDO) is fully biodegradable and is considered a promising material for future biomedical applications [211,212,213].

Unlike bio-derived, non-biodegradable plastics, aliphatic polyesters based on 2,5-furandicarboxylic acid have no commercially available petrochemical counterpart. Nevertheless, this class of polymers is emerging as a “sleeping giant” in the bioplastics market. These polyesters, known as poly(2,5-alkylenefuranoates), are synthesized via polycondensation between an alkylene glycol and 2,5-furandicarboxylic acid (FDCA) [214]. Environmental concerns have led to renewed interest in products derived from renewable resources. The main groups are the following: (i) agropolymers (e.g., polysaccharides, proteins) and (ii) biopolyesters (biodegradable polyesters) such as polylactic acid (PLA), polyhydroxyalkanoate (PHA), and both aromatic and aliphatic copolyesters [215].

Biodegradable nonwoven materials can be utilized in nearly all traditional nonwoven applications. In the sanitary and medical industries, a hair cap made of a nonwoven material based on thermoplastic poly(L-lactic acid) resin demonstrated good hair-retention properties, as described in Japanese Patent JP 2002345541 [216]. A breathable, biodegradable/compostable disposable personal hygiene product was produced from Bionolle 3001 nonwovens, as described in WO Patent 2002053376 and JP Patent 2002035037 [217,218].

Natural coconut fibers (coir) were used in biodegradable erosion control mats developed by Landlok for use in the geotextile industry. In the automotive industry, most European manufacturers already use natural fiber-based car interiors. In Germany alone, 3630 tons of flax, sisal, and jute were used in car interiors in 1996, rising to 11,800 tons by 1999. Although the absolute production volume remains modest, the average annual growth rate of approximately 50% is promising [219].

Nonwoven materials made from kenaf fibers offer good sound insulation properties for vehicle interiors [220]. Yachmenev and colleagues reported that various moldable nonwoven cellulose-based composites for automotive applications, with excellent thermal insulation properties, were manufactured using kenaf, jute, flax, and cotton waste in combination with recycled polyester and low-grade polypropylene [221]. In the filtration industry, biodegradable PLA-based nonwoven materials were used in products such as trash bags and sink drain filters [222]. Additionally, biodegradable pleated filter materials and filter blocks were developed for air purification and liquid filtration [223].

It is worth emphasizing that among all the biodegradable polymers synthesized from renewable resources, PLA is undoubtedly the most promising polymer to date [224]. Derived from 100% renewable sources such as corn and sugar beet, these polymers have recently become commercially viable alternatives to traditional polyolefin-based materials [225]. The biodegradation of polyolefins in the presence of starch is a complex process, heavily influenced by various factors, including the oxidation reactions of carbogenic macromolecules [226]. PLA is recyclable and compostable [227], and its physical and mechanical properties can be modified through polymer architecture [228,229,230].

Bio-based PDO (1,3-propanediol) is produced by the microbial fermentation of glucose using a process developed by DuPont and Genencor in 2003 [231]. This biotechnological route enables the production of high-purity and economically competitive PDO, facilitating its broader application in biopolymers and other chemical products [232].

Bio-based polyamides (BioPAs) are condensation polymers featuring repeating amide bonds in their molecular chains, which enable interchain hydrogen bonding, leading to an ordered microstructure and high crystallinity. This accounts for their strong mechanical properties, such as good impact resistance, high hardness, and excellent abrasion resistance. Polyamides may be synthesized via the condensation of diacids and diamines or from a single repeating unit containing both carboxylic and amine functionalities [233].

Though bio-based PET (Bio-PET) and polytrimethylene terephthalate (Bio-PTT) are only partially derived from biological sources due to their petrochemical-based terephthalic acid (TA), recent advances have made it possible to produce bio-based TA from various intermediates such as isobutanol, limonene, muconic acid, and furan derivatives like hydroxymethylfurfural [234].

Although PET is generally regarded as non-biodegradable and non-compostable, some natural biodegradation has been reported due to enzymatic activity. A newly identified bacterium has demonstrated the ability to utilize low-crystallinity PET as a carbon source through the action of PET-hydrolyzing enzymes such as PETase, which may pave the way for new biorecycling methods [235].

For convenience, the key points are summarized and provided in Table 3.

## 4. Applications in Various Sectors

### 4.1. Medicine, Tissue Engineering, and Scaffolding

There is a growing interest in biodegradable materials for applications in medicine and other sectors of the national economy. Synthetic biodegradable polymers are widely used in medicine for developing controlled drug delivery systems, surgical sutures, and orthopedic devices (such as screws, pins, and rods), as well as for the fabrication of nonwoven materials and matrices for tissue engineering. The most in-demand polymers for biomedical applications include aliphatic polyesters of α-hydroxy acids, such as polylactide (PLA), polyglycolide (PGA), poly(ε-caprolactone) (PCL), polydioxanone (PDO), and their copolymers. Materials derived from chitin offer promising advantages due to their enhanced biodegradability, making them attractive for medical applications [200].

Polyglycolic acid (PGA), due to its strong mechanical properties and high biodegradability, is especially suitable for use as absorbable surgical sutures. In 2010, the market for biomaterials for sutures was valued at GBP 1.1 billion [236]. PGA and its copolymers represent the largest segment by volume in the medical suture industry among commercial biopolymers [237]. Glycolide is often copolymerized with L-lactide to produce a polyglycolide-co-lactide (90:10) copolymer (PGLA). PGA-based materials are widely used in medical procedures including screws, nails, bone fracture treatments, and internal organ repairs [238]. PGA accounts for less than 1% of the biopolymer market. Since it is used solely in medical applications, where it biodegrades in the body, it does not require collection or recycling [118]. PLA and PGA are among the few synthetic polymers approved for clinical human use [239]. Currently, they are used as surgical sutures [240] and in controlled-release drug delivery systems [241], among other medical and pharmaceutical applications [242].

Adjusting the molecular and supramolecular architecture of biodegradable polymers enables the customization of their physical, chemical, and mechanical characteristics, along with the regulation of their degradation rate over time. This allows for the selection of optimal compositions and structures for the development of a wide range of biomedical devices. Incorporating various functional fillers, such as calcium phosphates, into the material structure enables the creation of bioactive composite materials with enhanced mechanical properties [243,244].

Techniques like electrospinning and lyophilization are utilized to fabricate finely dispersed biomedical materials, particularly for applications in regenerative medicine. Biocompatible materials are currently in high demand for general and cardiovascular surgery and the fabrication of pins and stents, vascular prostheses, artificial heart valves, and extracorporeal circulation systems, as well as for orthopedics, traumatology, and dentistry. They are also essential in cellular and tissue engineering, including reconstructive surgery, the development of artificial organs and tissues, and/or the restoration of the functions of damaged organs [245,246].

**Figure 4:**
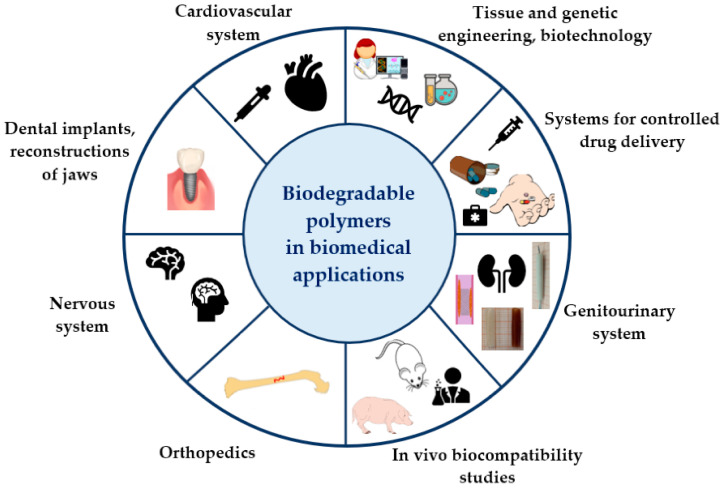
Schematic comparison highlighting the benefits of biodegradable polymers [247]. (This Figure is available as open access).

Figure 4 displays a schematic comparison highlighting the benefits of biodegradable polymers in contrast to non-biodegradable materials, particularly emphasizing their fundamental advantages.

Furthermore, nanostructured, biodegradable, and biocompatible polymers are increasingly being used in the development of next-generation drug delivery systems. Manipulating the molecular structure and supramolecular organization of polymers makes it possible to regulate not only the physicochemical properties and resorption time of materials and products but also their interaction with the living tissues of the patient [248].

Materials based on lactic and glycolic acids, as well as others such as poly(dioxanone), copolymers of poly(trimethylene carbonate), and homopolymers and copolymers of poly(ε-caprolactone), have been approved for use in medical devices [249]. In addition, poly(lactic-co-glycolic acid) copolymers offer a wide range of degradation rates—from days to years—achieved by varying the monomer ratio [250]. Numerous biodegradable polymers have been explored as scaffolds for tissue engineering. Porous polymer scaffolds promote tissue regeneration by providing a temporary framework for cell attachment and matrix synthesis [251].

Microbial synthesis has also proven effective in producing poly(β-hydroxybutyrate) (PHB), a strong and biodegradable PHA biopolymer. The bacterial synthesis of PHB depends on the availability of carbon-rich precursors used as a food and energy source. Unlike other microbially synthesized biopolymers, PHB is suitable for high-strength applications due to its mechanical properties, which closely resemble those of petroleum-based polymers like polypropylene. Its main limitation is its cost—approximately nine times higher than other biopolymers—driven by the market price of carbon-rich feedstocks. This cost challenge has been addressed through the use of agricultural waste, such as rice and sorghum processing residues [252].

PLA/layered silicate nanocomposites have been extensively studied by Sinha Ray and colleagues [253,254] and other researchers [255,256]. They successfully developed a series of biodegradable PLA nanobiocomposites mainly through PLA melt extrusion, often with organically modified montmorillonite (O-MMT), aiming to exfoliate nanofillers within the matrix. The production of biodegradable nanoporous polymer foams using PLA/layered silicate nanocomposite technology has also been described [254,257], using supercritical carbon dioxide as the foaming agent, with silicate acting as a nucleating site. Porous PLA structures can also be fabricated via co-continuous phase structures and the selective extraction of one component [258].

Extensive research has been carried out on PLA and its copolymers for biomedical applications in absorbable medical implants [259,260,261,262] in the form of rods, plates, screws, fibers, sheets, sponges, and microspheres for drug delivery systems [263], or as films and foils for wound treatment and agricultural use (e.g., mulching, slow-release fertilizers, and pesticides) [264].

Biodegradable polymers are widely used for the production of resorbable medical devices and organ/tissue prototypes. A key challenge is the modification of biodegradable polymers to introduce new functional properties. The clinical “gold standard” in regenerative therapy for wounds and burns is autologous skin grafts. These do not trigger immune responses and inherently possess the necessary biological and physicochemical characteristics. Both natural and synthetic polymers can serve as scaffolds for skin equivalents, but biodegradability is essential to enable scar-free tissue replacement. The biomechanical properties of the scaffold significantly influence fibroblast proliferation: more robust matrices tend to resist contraction, which enhances cell viability [265,266].

Nano- and microfibrous structures made from biodegradable polymers offer numerous advantages for biomedical applications, including tissue engineering systems. In one study, a mixture of poly(lactic acid) (PLA) and gelatin (GEL) conjugated with epidermal growth factors (EGFs) was used to electrospin nanofibrous scaffolds for their potential application in diabetic wound care [267].

Porous biodegradable polymer scaffolds are promising matrices for the reconstruction of damaged tissues and organs. Several methods have been described for the fabrication of such materials. Many of these methods involve polymer dissolution and salt leaching [268,269]. However, the porosity of PLA-based polymers has also been achieved through methods such as freeze-drying emulsions [270], gas-foaming agents [271], high-pressure gas saturation [272], phase inversion via immersion precipitation [273,274,275], thermally induced phase separation (TIPS) [276], and polymer blending followed by extraction [277,278].

Microfibrous structures of nonwoven, biocompatible, biodegradable polymers that release medical agents upon contact with the wound surface satisfy most requirements for wound and burn dressings. Electrospinning from polymer solutions is currently the most effective method for producing nonwoven materials made of micro- and nanofibers. This method allows for the fabrication of highly porous materials with unique filtration properties. Moreover, depending on the application, materials can be produced with uniformly or superficially distributed fillers within the fibers [279,280].

Designing or selecting porous scaffolds for tissue engineering involves a thorough understanding of how the scaffold’s three-dimensional microarchitecture influences both its biological integration and mechanical performance. The body’s response to an implanted scaffold is shaped by numerous parameters, including the choice of biomaterial, its degradation characteristics, and its structural design at the microscale level [281,282,283]. Biodegradable polymers offer several key advantages over permanent solid implants, particularly in medical and clinical settings. These benefits extend beyond functionality, including cost-effectiveness and improved patient experience. For instance, unlike metallic implants, biodegradable alternatives eliminate the need for secondary surgical procedures to remove the device after healing is complete [284,285,286].

An implantable device that does not require surgical removal offers additional benefits. For instance, a broken bone fixed with a rigid, non-biodegradable stainless-steel implant tends to refracture upon implant removal due to load shielding. However, a biodegradable polymer implant can be designed to degrade gradually, transferring the load to the healing bone over time [287]. Another exciting application of biodegradable polymers is in drug delivery—either as standalone delivery systems or integrated into medical devices. In orthopedic applications, for example, the delivery of bone morphogenetic protein can accelerate fracture healing [288], and antibiotic delivery may help prevent postoperative osteomyelitis [289].

To improve the flexibility and processability of polyhydroxybutyrate (PHB), researchers have examined the incorporation of biodegradable, low-molecular-weight, and non-toxic plasticizers such as dibutyl sebacate (DBS), dioctyl sebacate (DOS), polyethylene glycol (PEG), Lapro1503 (L503), Lapro15003 (L5003), and polyisobutylene (PIB), a non-polar polymer. These additives have been studied in concentrations reaching up to 50 wt%. Within the range of 15–20 wt%, the plasticizers remained highly compatible with PHB, resulting in homogeneous, single-phase blends. However, exceeding this concentration threshold typically led to a decline in system integrity due to over-plasticization. Many of these plasticizers were found to effectively lower the crystallization temperature while enhancing the material’s mechanical performance. Additional plasticizers mentioned in the literature include dodecanol, lauric acid, tributyrin, and trilaurin [290].

Historically and currently, the industrial use of PHA-based bioplastics has concentrated heavily on biomedical applications due to the excellent biocompatibility and biodegradability of PHAs. Products such as artificial skin, heart valves, vascular grafts, bone graft substitutes, scaffolds, and drug delivery systems have all been developed using PHAs [291]. Their biodegradability, compatibility with biological systems, and production from renewable feedstocks make PHAs suitable for various medical purposes, including surgical sutures, implantable devices, artificial blood vessels, tissue scaffolding, and controlled-release drug carriers [292,293]. Recognizing their commercial promise, numerous companies have launched PHA production initiatives at both pilot and industrial levels. Currently, close to 20 companies across nations including the United States, Austria, the United Kingdom, Germany, Italy, Japan, Brazil, and China are actively engaged in PHA manufacturing and commercialization [294].

The use of bioplastics containing polyhydroxyalkanoates (PHAs) for the production of small-scale, high-value biomedical devices is becoming a reality. However, even for these applications, more efficient and cost-effective processes must be developed for the production, extraction, purification, and enhancement of PHA material properties [295].

Biomedical applications of polylactic acid (PLA) include the development of scaffolds [296], biodegradable/resorbable fibrous medical textiles [297,298], orthopedic screws [299], biocomposite materials [300,301], and sutures [302,303]. In addition, low-molecular-weight PLA is used for tissue engineering [304,305,306].

Photo-crosslinked synthetic biodegradable polymer networks are particularly promising for biomedical applications such as drug delivery, cell encapsulation, and tissue-engineering scaffolds. By modifying the architecture, chemistry, degree of functionalization, and molecular weight of macromer precursors, networks with a broad range of physicomechanical properties, crosslinking densities, and degradation characteristics can be developed for various applications. These networks are easily fabricated and can incorporate a wide range of biologically active substances and cells. Moreover, the spatial and temporal control of crosslinking during additive manufacturing enables the fabrication of complex, structured networks. Photo-crosslinked networks have been used in drug delivery systems to provide controlled, prolonged release. Additive manufacturing methods such as extrusion-based techniques and stereolithography have been employed to prepare photo-crosslinked tissue engineering matrices. These methods allow precise control over pore size, architecture, and mechanical properties. Specifically, a variety of resins based on biodegradable photo-crosslinkable macromers have been developed for stereolithography [307].

The key points of this chapter are summarized in Table 4.

### 4.2. Edible Packaging and Films

A wide range of biopolymer-based materials have demonstrated potential for use in food packaging applications. The list of these materials includes (but is not limited to) the following: polylactic acid (PLA), sugar palm nanofibrillated cellulose (SPNFC), composites made from coffee grounds, and PBAT, as well as materials derived from blueberry agro-waste and corn starch. Innovations such as photobleaching have been used to alter the microstructure of starch-based and blueberry-derived biopolymers, leading to the creation of intelligent packaging systems capable of monitoring food quality [308]. Since the 1970s, starch-based biodegradable plastics have been under active investigation worldwide. Advances in processing have enabled the commercial-scale production of extruded films and molded products containing over 50% starch. To address their inherent sensitivity to moisture, these materials are often laminated with polyvinyl chloride to enhance performance, which is not itself biodegradable [309]. Bioplastics are primarily produced from renewable organic feedstocks, including polysaccharides (like starch, cellulose, lignin, and chitin), proteins (such as casein, gelatin, and gluten), and lipids derived from both plant oils and animal fats [310].

Enhancing mechanical strength often compromises biodegradability, necessitating blending with other polymers [311]. Some biologically derived precursors, such as cellulose acetate, possess high tensile strength (~90 MPa) but are not biodegradable [312]. The polymer industry faces the critical task of developing packaging materials that preserve product integrity throughout its life cycle and are capable of biological or physicochemical degradation post-use under environmental exposure [313]. Such packaging should decompose into harmless substances such as water and CO_2_, minimizing environmental impact. These materials often incorporate plant-based components, such as polysaccharides, grain-processing waste, and various types of starch [314]. Recent environmental concerns have spurred interest in biodegradable packaging materials. Such materials are often derived from agricultural biopolymers capable of forming coherent, continuous matrices. Initially, most research focused on cellulose and starch due to their abundance and low cost. However, their poor elasticity limits their application [315,316].

Over the past twenty years, the production of plastic products for packaging goods and food—such as polyolefins—has grown, leading to a corresponding increase in plastic waste. This is due to the tendency of such materials to accumulate in nature as a result of their superior mechanical strength and resistance to chemical, atmospheric, and biological degradation [317,318]. In the last decade, there has been a heightened interest in using commercially available proteins to prepare biomaterials, especially films [319,320].

**Figure 5:**
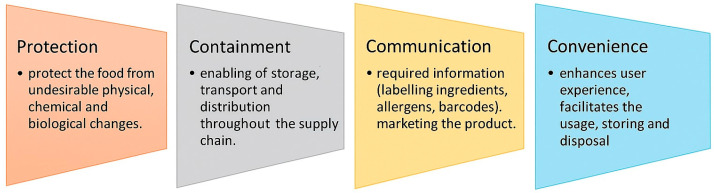
Roles and functions of packaging. Reproduced from [321]. (This Figure is available as open access).

Biodegradable plastics are primarily used in the food packaging and agricultural industries. In the food sector, packaging serves multiple roles, as demonstrated in Figure 5.

Convenience in food packaging enhances the user experience, encourages repeated purchase, and can differentiate a product in a competitive market. Packaging should make the product easy to use, handle, open, reseal, store, and dispose of. Examples of such packaging features include the following: resealable zip-locks for freshness, microwavable containers for quick heating, portion-controlled packaging for on-the-go consumption, easy-tear seals or ergonomic designs for elderly or disabled users, etc. [322,323].

Certain plant proteins demonstrate useful properties for preparing packaging biomaterials, such as network-forming ability, plasticity, and elasticity. Research on the film-forming potential of various plant proteins has primarily focused on soy proteins. Edible films have been made from isolated soy protein (ISP). Alkaline treatment increased the elongation percentage. Water vapor permeability (WVP), oxygen permeability (O_2_P), and tensile strength (TS) were not significantly affected by the alkaline treatment. A minimum pH of 8 was required when using ammonium hydroxide as an alkaline source to produce a satisfactory film, and pH levels above 8 did not further enhance the film properties. The exceptionally low oxygen permeability values of ISP films make them promising for protecting food products from oxidative spoilage [324]. Synthetic polymers play a key role in many industrial sectors, especially in the packaging industry [325].

Chitin and chitosan blends are gaining increasing importance as bases for the production of biodegradable packaging films and textile fibers. Chitosan-based films are formed from acetic acid solutions, with their solubility and swelling behavior regulated by crosslinking the chitosan with glutaraldehyde or oligomeric diepoxides [326].

Crosslinking polymers and the graft copolymerization of natural polymers with synthetic monomers are additional valuable approaches in creating biodegradable packaging films. A further advantage of such materials is that upon biodegradation, decomposition, or composting, they can act as fertilizers and soil enhancers, thereby contributing to improved crop yields. Although biopackaging is relatively expensive, it represents the future of packaging—particularly for several types of value-added food products [327]. Composites are being developed for packaging applications using polyethylene and polypropylene waste mixed with residues from the flour milling, starch production, sugar processing, and confectionery industries [328]. Chitin and chitosan have also been utilized as fillers [329,330].

PLA is inherently a polar material due to its repeating lactic acid unit. This high polarity imparts several unique characteristics, such as high critical surface energy, which ensures excellent printability. PLA is also used in agricultural films, compostable garbage bags, thermoformed trays for fruits and vegetables, disposable plates and cups, toys, tableware, fiber composites [331], and layered silicate nanocomposites [332,333,334,335]. Commercially available PLA packaging can exhibit superior mechanical properties compared to polystyrene and possesses properties more or less comparable to PET. Market studies indicate that PLA is economically viable for packaging applications and currently represents the largest market segment by volume for biodegradable packaging [336]. Bioplastics are considered highly significant for promoting sustainability, which encompasses the balance between the economic, environmental, and social aspects of business and can be applied across numerous industries.

Biodegradable packaging films are typically prepared by casting an aqueous solution onto a suitable base material, followed by drying. The choice of base material is crucial to facilitate the easy peeling of the film without tearing or wrinkling. Infrared drying chambers offer the advantage of speeding up the drying process [337,338]. An optimal moisture content of 5–8% in the dried film is desirable for easy peeling from one edge of the base material. Biopolymer films generally cannot be processed using blown-film extrusion as with synthetic polymers due to their lack of a defined melting point and tendency to degrade when heated. Film formation typically involves inter- and intramolecular associations or the crosslinking of polymer chains into a semi-rigid three-dimensional network that traps and immobilizes the solvent. The level of cohesion is influenced by factors such as the polymer’s structural characteristics, the choice of solvent, temperature conditions, and the inclusion of additives like plasticizers. In composite formulations or films, lipid components contribute to a visually attractive, glossy surface appearance [339,340].

Other polysaccharides—such as cellulose and chitosan—are also actively being developed as renewable, biodegradable raw materials for thermoplastics. Polymers obtained through the interaction of cellulose with epoxy compounds and dicarboxylic acid anhydrides completely degrade in compost within four weeks. Such materials are used to mold bottles, disposable tableware, and agricultural mulching films [35,341].

**Figure 6:**
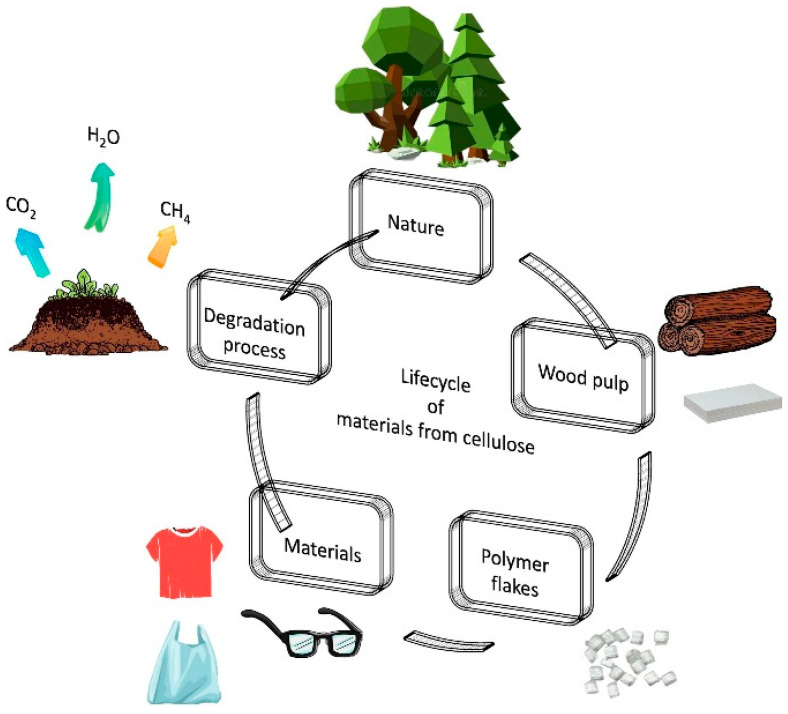
Illustration of the lifecycle stages of materials derived from cellulose [342]. (Permission to use was granted by Elsevier).

The lifecycle of cellulose-derived materials is depicted in Figure 6, illustrating the transformation from natural resource to final product through a series of stages. It begins with the use of a tree as the primary raw material, emphasizing the renewable nature of cellulose sourcing. The next step involves extracting wood pulp, which serves as a key intermediate and the foundation for producing cellulose-based polymers. This pulp is then processed into polymer flakes, a critical phase in the development of these materials. Following this, a deflation step is carried out, allowing for the controlled breakdown of polymer flakes into smaller, usable components. The cycle concludes with the material undergoing biodegradation, highlighting its environmentally friendly end-of-life pathway [342].

To reduce the production cost of biodegradable household materials (such as packaging, agricultural mulch films, and garbage bags), it is recommended to use unrefined starch mixed with polyvinyl alcohol and talc [343]. Temperature-resistant multilayer packaging materials are produced using cellulose films bonded with starch to fat-resistant paper approved for food contact. This type of packaging is suitable for baking foods in electric or microwave ovens. Natural proteins have also attracted the attention of biodegradable plastics developers. For example, zein—a hydrophobic protein—is used to produce films for wrapping moist foods and manufacturing food containers [344,345].

The only type of household waste that does not require separate collection and special disposal conditions is biodegradable edible packaging. This includes films and sheets approximately 250 µm thick, as well as bags, soft gel capsules, and hard coatings on tablets, that are food-grade [346,347,348,349]. These materials are made from renewable sources and thus degrade faster than synthetic materials. In addition to polymers and waxes, other key ingredients—such as glycerol, propylene glycol, and sorbitol—are used to enhance the flexibility, strength, and viscosity of films and coatings. Aqueous alcohol solutions are commonly used as solvents in film-forming compositions [350,351].

Biodegradable films and coatings are widely used for fruits and vegetables to reduce moisture loss, prevent weight reduction, enhance appearance, and reduce gas exchange rates [352,353,354]. The organoleptic properties of packaged food products are improved with the use of edible films, especially when they include components such as flavorings, colorants, and sweeteners. These films can carry functional ingredients that extend shelf life by preventing microbial spoilage, rancidity, enzymatic browning, and the development of off-flavors. Functional additives include antioxidants, nutraceuticals, spices, and natural colorants [355,356].

Natural polymers such as starch derivatives, gelatin, cellulose, and sodium/calcium alginates derived from brown seaweed are commonly used as the base for film-forming edible coatings. These prevent moisture loss, regulate oxygen and carbon dioxide exchange, provide structural integrity, and help retain the quality and nutrients in packaged food [357]. Edible films made from natural polymers also exhibit a high sorption capacity. Once ingested, they can remove harmful compounds such as metal ions and radionuclides, acting as detoxifiers. When flavorings and colorants are added to edible polymer shells, they can enhance or modify the taste and aroma of the food product. This is especially valuable for foods with a reduced fat or sugar content or those enriched with plant proteins. Edible films can also enrich food with minerals, micronutrients, and vitamins. Moreover, such packaging simplifies food consumption by eliminating the need to unwrap, reducing content loss [358].

Employees of the Borisov Polymer Packaging Plant “Polimiz,” in collaboration with scientists from Belarusian State University, developed an edible film based on starch and food polymers in water. This film not only extends product shelf life and enhances consumer appeal but is also easily digestible and even has potential preventive health benefits [359]. This initiative has helped Belarus manage its resources sustainably and reduce household waste. A thin layer of this edible film can be applied to protect food from dust and preserve freshness. Biodegradable polymers are also essential in waste reduction strategies [360].

There is growing interest in developing bioplastic products based on canola protein isolates (CPIs). CPI-based films have great potential for food packaging. They can be applied between food layers or on food surfaces to control moisture, oxygen, CO_2_, aroma, and lipid migration. These films can also be heat-sealed to form sachets, pouches, or bags for storing dry goods. However, their poor mechanical strength and low water vapor resistance remain challenges. Improvement methods include protein denaturation, blending with biodegradable/synthetic polymers, and adding nanoclay or fibers [361].

### 4.3. Agricultural Waste as Feedstock for Bioplastic Production

Agricultural waste materials, including grape pomace, tomato pomace, pineapple, citrus peels (orange and lemon), rice husks, sugarcane bagasse, palm oil fibers, wheat straw, and other easily accessible resources, serve as carbon-rich feedstocks for biopolymer production via microbial, biopolymeric, and chemical processes [362]. Examples of the resulting biodegradable materials are composites of low-density polyethylene (LDPE) and high-density polyethylene (HDPE) filled with sunflower seed husks [363].

The choice of suitable agricultural waste depends on several important factors: (i) the starch content; (ii) levels of cellulose, lignin, and hemicellulose; (iii) bioavailability and potential effects on agricultural supply chains and food security; (iv) complexity of the synthesis methods and the targeted material properties; and (v) biodegradability [364,365,366].

Plant-derived cellulose is usually combined with other polymers such as lignin, hemicellulose, and pectin, while bacterial cellulose is extremely pure. The unique properties of bacterial cellulose are attributed to its ultrafine nanofibrils forming a three-dimensional network structure [111,367]. Oils are also excellent carbon sources for bioplastic production. Various oils have been investigated, including cottonseed oil [368], soybean oil [369], crude palm kernel oil, jatropha oil, crude palm oil, palm olein, corn oil, and coconut oil [370]. Lignocellulosic biomass is another promising resource for bioplastic production, as it circumvents the use of food crops [371,372]. The recyclability of biodegradable polymer matrices and their cellulose-reinforced composites has also been studied, demonstrating potential integration into plastic recycling systems [373].

Coffee grounds contain cellulose and hemicellulose (about 20%), pectin, lignin, microelements, and proteins. Due to their high thermal degradation temperature (approximately 285 °C), they can be processed using conventional composite manufacturing techniques. The biodegradability of coffee grounds is attributed to their cellulose content, as well as the presence of trace elements and proteins, whose biodegradation mechanisms are similar to those found in wood flour [374,375,376,377]. Lignocellulosic fibers are extracted from plants such as curaua, pineapple, sisal, and jute [378].

Agricultural waste is a major source of raw materials used in the production of bioplastics, plasticizers, and antioxidant additives [379]. These wastes are a rich source of polysaccharides, which are crucial precursors for the development of natural plasticizers [380]. Plasticizers primarily function to increase the elasticity and mechanical strength of biopolymers. However, the effectiveness of plant-based polysaccharide plasticizers compared to glycerol and its synthetic counterparts has not been definitively established [381].

Merlot grape pomace is the primary agricultural waste in winemaking. Rather than discarding this waste, it can be a viable source for composites produced through solvent extraction (SE) and pressurized liquid extraction (PLE). Extracts obtained from SE and PLE methods are blended with commercial-grade PHAs to form a matrix. In the final production stage, the biopolymer is mixed with poly(3-hydroxybutyrate-co-3-hydroxyvalerate) (PHBV), a copolyester containing hydroxyvaleric acid, to form active biocomposites. These biocomposites demonstrate higher tensile strength compared to pure biopolymers or isolated matrices. Solvent-extracted biomaterials resulted in reduced tensile strength but slightly improved elongation at break. The data also indicate that the extraction method influenced the mechanical properties—SE proved to be a more practical method than PLE. Sugar beet agro-waste is also a promising source for biocomposites due to the presence of carboxyl groups in the dried pulp [382].

Agriculture represents a high-potential market for nanocomposite bioplastics derived from bacterial biomass containing PHAs. One notable application is the replacement of black plastic mulch, used for weed suppression, moisture retention, and soil warming for early planting. Low-cost crude bioplastic production is achievable via mixed microbial cultures under non-aseptic conditions. Organic acids produced through the acidogenic fermentation of municipal solid waste (MSW) serve as a dominant carbon source for PHA biosynthesis [383]. Primary agro-waste sources include grape stems, olive pits and pomace, and citrus peels (lime and lemon) [384,385].

Unlike renewable sources obtained from cultivated plants, agricultural waste is derived from post-harvest residues and food processing byproducts, such as coconut shells [386], potato peels [387], fruit peels [388], and fruit seeds [389]. Waste from agriculture, food, and biofuel production containing palm oil, seeds, fats, and used cooking oils—as well as glycerol from fat hydrolysis and biodiesel production—can be used for the economical production of PHAs, either through the chemical hydrolysis of long-chain fatty acids and glycerol or by the direct biotransformation of triacylglycerides [390,391].

The use of natural additives in bioplastics is a relatively new development, whereas commercially available bioplastics often contain synthetic additives. In addition to adding natural elements, UV-induced degradation is inhibited by using maleic anhydride treatment, reactive mixing, and graft copolymerization throughout the synthesis process [392]. Essential nutrients like nitrogen, phosphorus, sulfur, iron, and trace elements can also be supplied from organic waste. However, the most critical factor in bioplastic production remains the cost of carbon and energy sources, often derived from the organic fraction of municipal solid waste (MSW). Proper MSW management for PHA production via mixed cultures represents a key ecological and economic challenge [393]. Biodegradable polymers are also produced through the activity of microorganisms, such as Gram-negative and Gram-positive bacteria, in the presence of carbon-rich materials like agricultural waste. The bacterial synthesis of polymers is typically initiated by pH changes and the limited availability of key nutrients such as phosphorus and nitrogen [394], as well as the composition and type of the microbial culture and growth medium [395].

### 4.4. Role of Biopolymers in Construction

The application of biopolymers in construction depends on reinforcing materials such as carbon nanotubes (CNTs), carbon nanofibers (CNFs), nanocellulose, cellulose, lignin, hemicellulose, and α-cellulose microfillers derived from agricultural waste. The reinforcement of biopolymers is essential because these materials are highly permeable to water and are biodegradable. Progress in materials science and nanotechnology has enabled the creation of innovative applications within the construction industry. Enhancing biopolymers by incorporating cellulose nanofibers (CNFs) and carbon nanotubes (CNTs) has increased the effectiveness of polymers derived from rice husks for use in building materials [396].

Composites made from α-cellulose microfillers and epoxy matrices have been used in construction to replace wood and substitute internal metal door panels in BMW and Mercedes-Benz vehicles. These α-cellulose microfillers are synthesized from agricultural waste such as date seeds, robusta coffee grounds, coconut shells, wood, oil palm shells, walnuts, hazelnuts, and red empty coconut fibers [397].

Crude bioplastics containing PHAs (polyhydroxyalkanoates) can serve multiple purposes in both construction and agriculture. In the construction industry, bioplastic foam containing PHAs can be used to manufacture foam insulation panels, silt and dust barriers, non-structural elements such as partition walls, and temporary structures [398].

The environmental benefits of producing and using crude bioplastics from the organic fraction of municipal solid waste include the following: (1) reducing the volume of waste sent for incineration, (2) decreasing the amount of ash requiring landfilling, and (3) enabling the use of seawater for waste separation, conserving freshwater resources [398].

The construction industry shows a growing trend toward the use of biodegradable materials and biopolymers [399]. Traditionally, almost all construction activities have relied on wood and other cellulose-based natural materials, which remain among the most commonly used biodegradable polymeric materials. PHA-containing bioplastic foam is an innovative, environmentally sustainable construction material that degrades rapidly in landfills or can alternatively be composted [398].

## 5. Starch-Based Bioplastics and Their Production Methods

### 5.1. Starch-Based Bioplastics

Starch remains a widespread, low-cost raw material in biomaterial development. Starch-based bioplastics can be produced by blending with synthetic polymers. Starch-based materials are garnering increasing interest due to their complete and relatively rapid biodegradability, low cost, and widespread availability from renewable sources [400,401,402]. The development of biodegradable starch-based materials generally follows two main strategies: (1) blending granular starch with synthetic polyolefin plastics such as polyethylene and polypropylene [402,403,404]; and (2) creating thermoplastic starch blends with natural and synthetic biodegradable polymers [405,406]. The increasing demand for biodegradable starch-based materials is driven by the global issue of petroleum resource depletion and the need to reduce the environmental impact of widespread petroleum-based polymer usage [407]. The polymer structure of starch used in packaging materials is susceptible to degradation by soil microorganisms and other environmental factors [408].

**Figure 7:**
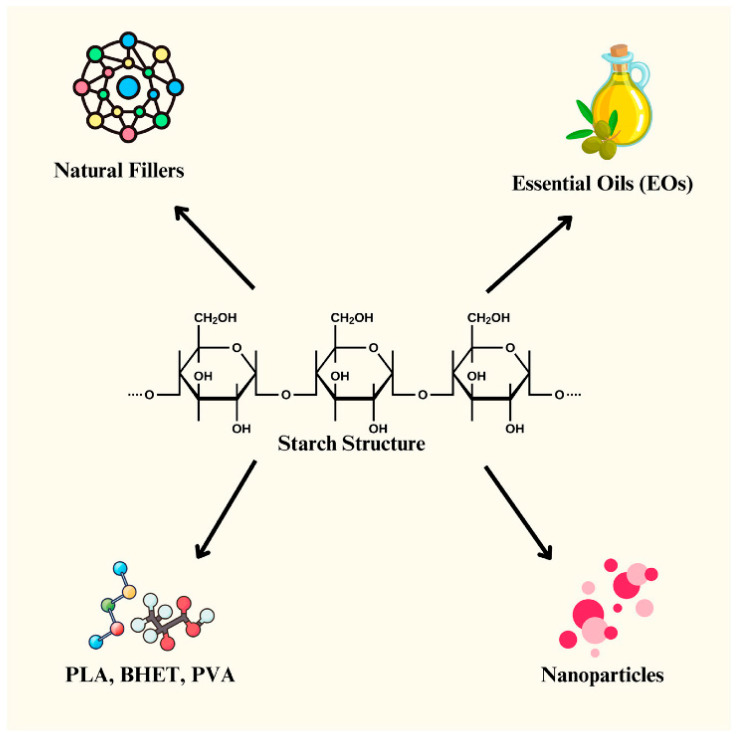
Molecular configurations of starch along with reinforcing fillers and polymer blend components [409].

A higher amylopectin content in starch contributes to increased crystallinity, whereas amylose enhances tensile strength, reduces elongation at break, and results in a higher Young’s modulus. These characteristics make starch an attractive candidate for bioplastic production, owing to its biodegradability, renewability, and wide availability [409]. To enhance the mechanical and functional properties of starch-based bioplastics, researchers have increasingly incorporated additives including natural fillers, essential oils, nanoparticles, and polymer blends such as PLA, BHET, and PVA, as illustrated in Figure 7.

A key advantage of thermoplastic starch-based biodegradable polymers is their ability to rapidly decompose in natural environments, unlike conventional petrochemical-based plastics [410,411,412]. Among its many advantages, starch is widely used due to its low cost and abundance [413]. It is produced by many plants and stored as an energy reserve within plant cells [414]. Dual-origin starches are developed through chemical modification, derivatization, and cross-linking. In food applications, starch provides various physicochemical and functional properties such as composition, crystallinity, and gel-forming capabilities [415].

Starches from various sources—such as potatoes, barley, wheat, tapioca, and rice—have been explored as potential film-forming agents, making starch one of the most promising materials to replace conventional plastics in select market segments [416]. Rapidly biodegradable starch-based plastics are used for packaging items like biowaste disposal bags and thermoformed trays, agricultural applications such as mulch films and plant pots, and hygienic and cosmetic products [417,418].

However, native starch is not inherently thermoplastic due to strong intermolecular hydrogen bonding. Therefore, it must be processed into a thermoplastic material using plasticizers like glycerol and water, in combination with heat and shear stress [419]. The addition of urea and certain polyols can improve starch plasticization, resulting in high-quality films. Destructured starch, obtained through the disruption of its granular architecture and a loss of crystallinity, is a novel thermoplastic material being commercially developed. To enhance the compatibility between hydrophilic starch and hydrophobic polymer matrices, surface modifications such as silane treatments are employed [420]. Pro-oxidants may also be added to accelerate the oxidative degradation of synthetic polymers [421].

The study in [422] demonstrated that combining thermoplastic starch (TPS), polylactic acid (PLA), and cellulose nanofibers (CNFs) in green nanocomposite films significantly enhanced their tensile strength (up to ~37 MPa) and Young’s modulus (~630 MPa), along with the induction of reduced water vapor permeability—indicating strong promise for food packaging materials. Additionally, the authors of [423] reviewed starch-based nanocomposites and emphasized that starch is an abundant, renewable, low-cost, and fully biodegradable polymer. They highlighted the use of nanofillers—such as nanocellulose, nanoclays, and metal oxides—to improve mechanical strength and barrier performance and broaden their application potential in packaging, agriculture, and biomedicine. In 2021, starch-based blends accounted for ~16.4% of global bioplastic production. The authors of [424] go over strategies—blending with PLA, PVA, and PBAT and using nanofillers—to overcome water sensitivity and mechanical limitations, positioning starch composites as eco-friendly alternatives to petroleum-derived plastics.

By leveraging the unique properties of starch and synthetic polymers, it is possible to design composites for applications in biomedicine and environmental technologies. Examples of starch-based materials used in food packaging span a variety of formulations. One common approach involves blending starch with synthetic polymers such as polyethylene or polypropylene to enhance its mechanical strength and moisture resistance. Alternatively, starch is often combined with other natural polymers to produce fully biodegradable films with improved flexibility and functionality. In some cases, starch is processed through extrusion to create thermoplastic starch (TPS), which can be molded into packaging products that serve as sustainable alternatives to conventional plastics [425]. Thermoplastic starch is considered one of the most promising components for the production of affordable biodegradable materials [426,427]. One of the most recognized starch-based commercial products is Mater-Bi, developed by Novamont S.p.A. in Novara, Italy. It breaks down in soil within 60 days and does so without emitting any toxic byproducts [428].

Among the many naturally derived polymers, starch is particularly notable. It accumulates in various plant organs—tubers, seeds, stems, and leaves—and is characterized by high biodegradability and renewability. These traits have made it a primary feedstock for biodegradable material production [429,430,431,432,433].

Starch-derived products such as dextrins and glucose are widely used as fermentation medium components. Glucose can be fermented into lactic acid, which is then polymerized into polylactic acid (PLA) and copolymers. These materials are of great interest and in high demand for biodegradable plastic applications [434].

Starch is emerging as an eco-friendly alternative to petroleum-based polymers due to its low cost, biodegradability, and ability to form films via thermoplastic processing. Starch films have been produced through casting and thermal processing using thermoplastic starch. Numerous components have been incorporated into the starch matrix, and its processing parameters have been modified to improve film properties. When optimally processed, the resulting films are transparent, odorless, tasteless, and colorless, exhibiting good mechanical, barrier, and optical properties [435,436,437].

However, retrogradation and the high hydrophilic nature of starch films limit their practical applications. The incorporation of certain additives—such as lipids, other hydrocolloids, and reinforcing agents—can significantly address these limitations, resulting in more stable materials with improved properties. Nevertheless, most existing studies have relied on casting methods, which have limited industrial applicability. Therefore, further research using thermal processing is essential to optimize starch-based film formulations that are scalable for commercial production. In this context, analyzing nano- and microstructural changes in starch matrices depending on their composition and processing conditions and how these changes relate to the final film properties is crucial for formulation and process optimization [438].

Starch varies in its botanical origin—typically derived from potatoes, rice, wheat, and corn. Significant scientific efforts have been dedicated to developing biodegradable polymers to conserve petrochemical resources and reduce environmental damage. When such materials are stored under natural conditions, they undergo hydrolytic degradation and disintegration under exposure to light and ultraviolet radiation, fitting into the natural environmental cycle [439].

Starch content is one of the primary criteria in selecting agricultural feedstocks. A preference for high-starch crops often implies a trade-off with the crop’s growth rate. Similarly, a higher cellulose content enhances mechanical strength but reduces the biodegradation rate. Thermoplastic starch-based polymers are practical alternatives to petroleum-derived plastics due to their effective reinforcement capabilities, abundance, and tunable properties [440].

Currently, thermoplastic starch is the most widely used bioplastic. It is obtained either through enzymatic saccharification and microbial fermentation or by modifying the starch using hydrophilic plasticizers [441]. Recently, composite bioplastics made from tapioca starch and sugarcane bagasse fiber were investigated. Ultrasonication was found to improve their properties by enhancing tensile strength and reducing moisture absorption rates [442].

The key points of this subchapter are summarized in Table 5.

### 5.2. Production Methods

There are several methods for starch film production:

Casting is one of the most commonly used methods for producing starch-based films. This method includes the following: (a) dissolving the biopolymer in a solvent/plasticizer, (b) casting the solution into a mold, and (c) drying. The process requires starch gelatinization, which involves mixing starch with water (3–12%), followed by heating above the gelation temperature [380].

Dipping (Immersion): In this technique, food items or substrates are submerged in a film-forming starch solution for a set period, removed, and allowed to air-dry. This is widely used at the lab scale for its simplicity and uniform coating coverage. Variations like vacuum and multiple immersion cycles can further improve coating adhesion and thickness control [443,444].

Brushing/Spreading: This manual method involves applying a starch-based solution to a surface with a brush, roller, or spatula. It allows precise control over the amount and distribution of the coating, making it useful for packaging irregularly shaped food or when layering multiple coatings [445,446].

Spraying: A thin mist of starch solution is atomized over the food surfaces, resulting in even, lightweight coatings with minimal material usage. This method is scalable and suited for larger or continuous processing applications [447,448].

Extrusion (Thermoplastic Processing): In this industrial-scale approach, the starch and plasticizer are blended under high heat and shear in an extruder, forming a thermoplastic starch melt. The melt is then extruded and thermoformed into films. The process parameters—temperature, screw speed, and moisture—critically influence film quality [449,450].

Electrospinning/Electrostatic Spraying: Although less common, emerging techniques like electrospinning and electrostatic spraying use high-voltage fields to produce nanofibrous starch-based films with a high surface-area-to-volume ratio. These films demonstrate excellent functional properties but remain largely experimental [31,451].

## 6. Recycling and Disposal

Although biodegradation can be regarded as a form of recycling—sometimes termed “organic recycling” [452]—it is not primarily aimed at recovering plastic materials or monomers for reintegration into the plastics lifecycle. For example, as long as the material quality remains high, biodegradable plastics can be mechanically recycled either through primary recycling, in which the recycled plastic is reused for the same purpose as the virgin plastic, or through secondary recycling for less demanding applications [453].

When the material quality falls below a certain threshold, bioplastics can undergo chemical recycling to recover valuable monomers for use as building blocks in new polymers or specialty chemicals. Finally, when the material quality is too low for reuse, bioplastic waste may be biodegraded (when feasible) or subjected to quaternary recycling through incineration. Therefore, biodegradation should not be assumed as the default or best end-of-life strategy for biodegradable plastic waste. Instead, all recycling strategies should be considered to maximize the environmental benefits of these materials [454]. Most biodegradable vinyl polymers contain oxidizable functional groups, and catalysts are often added to accelerate oxidation or photo-oxidation [455].

As was stated earlier, biodegradable polymers can be classified as either natural or synthetic. Synthetic polymers present multiple benefits compared to their natural counterparts, such as the capability to customize a wider variety of properties, greater consistency between production batches, and a more dependable supply of raw materials that avoids challenges like immunogenicity [456]. Microorganisms primarily attack oxygen-containing bonds. Among these, ester groups are most susceptible to enzymatic degradation [457]. The rate of plastic biodegradation depends primarily on the structure of the polymer matrix and, secondarily, on the nature of its pro-oxidant additives [458,459].

Enzymes secreted by microorganisms may either be released externally to degrade the plastic surface or act internally by engulfing small oligomeric plastic fragments. Thus, two types of enzymatic degradation are recognized: exogenous (external) and endogenous (internal). For the same reason, polymer-degrading enzymes are categorized as extracellular and intracellular depolymerases [460]. To regulate the rate of biodegradation, a wide range of complexing agents are used, including deoxysuccinates, epoxides, and layered organosilicates [461,462].

A study of the technical challenges in bioplastic production revealed a complex entanglement within their niche markets and difficulties in penetrating the mainstream market. The growing problem of waste disposal and the high cost of pure substrates in the production of polyhydroxyalkanoates highlight the future necessity of upgrading waste streams from various industries to serve as feedstock for PHA production. In addition to low-cost carbon sources, efficient upstream and downstream processing and the recycling of waste streams throughout the process are required to sustain circularity in the overall system [463].

A crucial aspect of biodegradable packaging is its end-of-life management. One promising solution for managing polymeric packaging waste is the development of new biodegradable materials. The decomposition products of such materials pose minimal risk to both the natural environment and human health [464,465,466,467]. Another approach to solving the problem of plastic waste is the development of specific microbial mutations capable of breaking down synthetic polymers [35]. There are various end-of-life (EoL) processing options for biodegradable polymers, including home composting, industrial composting, chemical recycling, catalytic recycling, mechanical recycling, enzymatic depolymerization, and anaerobic digestion. The choice of EoL treatment depends on the type of precursor [468]. The rate of biodegradation also depends on the microbial strains used during microbial synthesis [469].

In summary, biopolymers are defined as polymers derived from renewable resources, as well as biodegradable polymers that may originate from fossil fuels [15]. The introduction of various modifying additives can significantly increase or decrease a polymer’s biodegradability. For example, ester-based plasticizers typically enhance the biodegradability of PVC. However, if a well-biodegradable plasticizer (e.g., dibutyl phthalate) does not diffuse adequately to the polymer surface, the overall biodegradability of PVC remains poor [35]. The COVID-19 pandemic gave rise to a new term—COVID-19 waste—referring to waste generated from the use of personal protective equipment (PPE), such as used disposable masks, gloves, and sanitizer bottles [470]. This situation can lead to disastrous consequences, including pollution, the contamination of the food chain, energy losses, economic damages, threats to biodiversity, and an increased environmental carbon footprint [471].

According to data published in the Environmental Science & Technology Journal, an estimated 129 billion masks and 65 billion gloves are discarded globally every month. As a result, there has been a significant increase in environmental pollution from nearly non-degradable plastics and polyethylene. To mitigate this, there is an urgent need to transition to the widespread use of biodegradable materials, particularly those that also offer additional functional properties [472]. To enable synthetic materials to degrade, various modifiers are used as catalysts to break carbon bonds and initiate the biodegradation process of synthetic polymers. Depending on the amount of modifier introduced, plastics can fully decompose within a time frame ranging from 3 months to 5 years. The concentration of the modifier directly affects the decomposition rate [28,473]. Special attention must be paid to the selection of such catalysts when producing packaging materials for dairy and long-shelf-life food products [474].

### 6.1. Recycling Options

There are two main recycling options, namely mechanical and chemical:

- Mechanical Recycling: This involves the physical processing of waste and is considered a primary approach for plastic recovery due to its relatively low cost, simple technology, and lower environmental impact compared to chemical recycling [475,476]. Though well established for conventional plastics, its application to biodegradable plastics requires caution. Most polymers in this category, including PLA, PHAs, and polyglycolic acid (PGA), are aliphatic polyesters and therefore thermally sensitive [111]. For example, PLA is primarily recycled through mechanical or chemical means or via industrial composting [477]. PLA and PGA are highly susceptible to thermal degradation, leading to discoloration and deterioration in their mechanical properties. This issue is exacerbated by their high hygroscopicity, where absorbed water promotes hydrolytic chain scission at elevated temperatures, thus accelerating thermal degradation. The precise drying of these materials before mechanical recycling is essential. Furthermore, effective drying may be complicated by contaminants such as paper, which can retain moisture [478]. The mechanical recycling process involves several stages, including waste collection, screening, manual and/or automated sorting, grinding, washing, drying, compounding/extrusion, and pelletizing. These stages may occur in varying sequences depending on the size, shape, and composition of the plastic waste [479].

**Figure 8:**
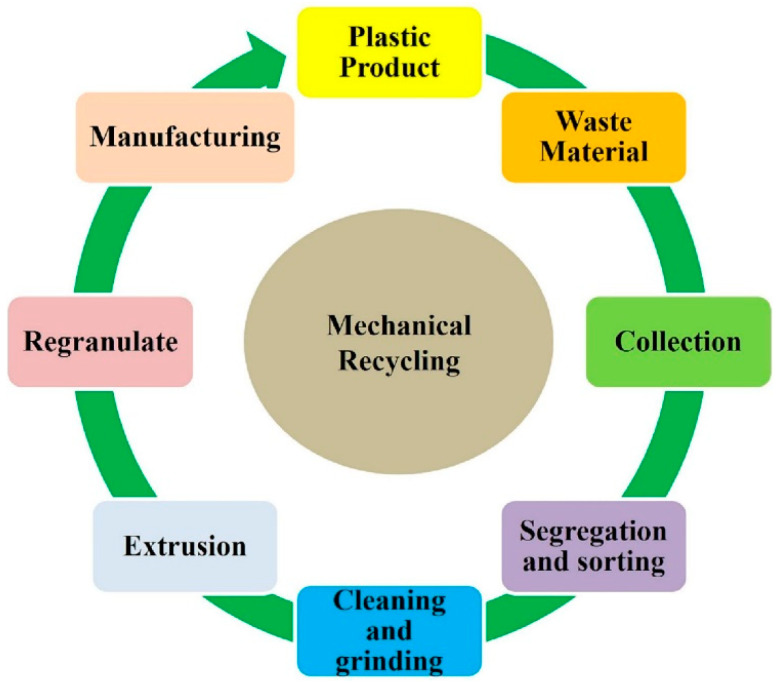
Stages of mechanical recycling. (Permission to use was granted by Elsevier).

Mechanical recycling is considered an environmentally friendly approach due to its low setup cost and operational simplicity, making it an increasingly attractive option for biopolymer recycling. The process typically includes several stages, such as the collection, separation, sorting, cleaning, drying, and shredding of waste materials (see Figure 8). Despite its advantages, the quality of its recycled materials often falls short compared to the original products. Importantly, the large-scale industrial mechanical recycling of biodegradable plastics has yet to be fully realized [480].

- Chemical Recycling: Also known as tertiary recycling, this is an emerging route that transforms waste into useful chemicals such as monomers and/or oligomers that can be reintroduced into the polymer value chain and reused for polymerization [481]. Although not yet prominent for biodegradable plastics, chemical recycling and solvolysis show economic and environmental promise. For example, recovering lactic acid from PLA waste via hydrolytic degradation may require less energy than producing it through biomass fermentation [111,482]. The tertiary recycling of biopolymers focuses particularly on aliphatic polyesters that can be depolymerized in a controlled manner, with the primary aim of conserving raw resources rather than merely reducing waste accumulation. Techniques include dry heat depolymerization (e.g., pyrolysis) and solvolysis methods (e.g., hydrolysis, alcoholysis) [483].

According to ISO Standard 14855-2, a material is considered biodegradable if 90% of its original mass is lost within six months at 59 °C [484]. In accordance with the European Waste Framework Directive, waste must be managed in line with a waste hierarchy, prioritizing (i) prevention, (ii) preparation for reuse, (iii) recycling, (iv) other recovery (e.g., energy recovery), and (v) disposal [485]. Effective recycling requires the use of efficient and economically viable technologies for sorting plastic waste to ensure high-quality and pure secondary raw materials [486,487].

### 6.2. Criteria for Compostability

Within the European Union, composting is primarily encouraged through the EU Landfill Directive [488], which urges member states to limit the quantity of biodegradable waste sent to landfills, and Directive 2008/98/EC, which promotes the segregation and proper management of biodegradable waste [489].

Polymer biodegradation is a complex process influenced not only by the chemical structure and properties of the polymer but also by environmental conditions. Key external factors include humidity, temperature, pH, light, and interactions with soil, including soil type [490].

Materials that do not meet the criteria for biodegradability may still be classified as compostable. Although every compostable plastic can break down naturally, not every biodegradable plastic qualifies as compostable [491]. The distinction lies in the mass loss under specific conditions [492,493].

A reduction in the molecular weight of macromolecules enhances their biodegradability. Crystallinity is another important characteristic; amorphous polymers biodegrade more easily than crystalline ones. Increased crystallinity and higher molecular weights reduce the rate of biodegradation. Conversely, branched macromolecular structures exhibit improved biodegradability [30].

One of the most essential factors in polymer biodegradation is the presence of compost (decomposing organic matter) [494]. Based on compost dependency, all biodegradable materials are categorized as either “compostable” or “non-compostable.” “Compostable” refers to plastics that degrade only in composting conditions but not in natural environments. Many consumers mistakenly equate “compostable” labels with “biodegradable,” leading to improper disposal and increased plastic pollution [495].

In order for a polymer to be classified as compostable, it must meet at least one of the following international standards [496,497].

- ASTM D6400 [ASTM D6400 12; Standard Specification for Labeling of Plastics Designed to be Aerobi-cally Composted in Municipal or Industrial Facilities. ASTM International: West Con-shohocken, PA, USA, 2012] (applicable to compostable plastics) or D6868 (designed for compostable packaging);
- European standard CEN EN 14995:2006 [EN 14995:2006; Plastics—Evaluation of compostability—Test scheme and specifications. European Committee for Standardization (CEN): Brussels, Belgium, 2006.], which applies to compostable plastics, or EN 13432:2000 [EN 13432:2000; Packaging—Requirements for packaging recoverable through composting and biodegradation—Test scheme and evaluation criteria for the final acceptance of packaging. European Committee for Standardization: Brussels, Belgium, 2000.], which covers compostable packaging;
- ISO 17088:2021 [ISO 17088:2021; Plastics—Organic recycling—Specifications for compostable plas-tics. International Organization for Standardization: Geneva, Switzerland, 2021].

The ISO 17088:2021 [ISO 17088:2021; Plastics—Organic recycling—Specifications for compostable plastics. International Organization for Standardization: Geneva, Switzerland, 2021] and ASTM D6400 [ASTM D6400 12; Standard Specification for Labeling of Plastics Designed to be Aerobically Composted in Municipal or Industrial Facilities. ASTM International: West Conshohocken, PA, USA, 2012.] standards follow the same testing protocol as EN 13432:2000 [EN 13432:2000; Packaging—Requirements for packaging recoverable through composting and biodegradation—Test scheme and evaluation criteria for the fi-nal acceptance of packaging. European Committee for Standardization: Brussels, Belgium, 2000.]. Unlike the other standards, the ISO standard covers not just plastic packaging but plastics more broadly. A polymer that meets any of these standards must accomplish the following:

1. Disintegrate rapidly during composting;
2. Biodegrade quickly under composting conditions;
3. Not diminish the quality or utility of the resulting compost, which must be able to support plant life;
4. Contain only minimal amounts of regulated heavy metals or other toxic substances.

The main distinction between biodegradable and compostable polymers is based on their rate of biodegradation, the way they break down, and their toxicity. Although all compostable polymers are by definition biodegradable, not all biodegradable polymers meet the criteria to be considered compostable [498].

## 7. Discussion

The growing interest in biodegradable polymers is a direct response to the environmental consequences of conventional plastic use, particularly their persistence in ecosystems and their associated waste management issues [9,28,150]. This review emphasizes the importance of molecular structure and processing methods in determining biodegradation rates and mechanical performance, which are often in tension [101,311]. A key observation across the literature is that enhancing mechanical strength—crucial for functional applications like packaging and medical devices—tends to reduce the rate of biodegradation, requiring trade-offs or polymer blending strategies [311,314].

Polylactic acid (PLA) and polyhydroxyalkanoates (PHAs) emerged as the most prominent and well-researched biodegradable polymers, owing to their renewability, the relatively low toxicity of their degradation products, and their suitability for both industrial and biomedical applications [111,138]. PLA, for instance, is readily synthesized via ring-opening polymerization, is industrially scalable, and offers desirable thermal and mechanical properties [98,110,119]. However, its hydrolytic degradation remains relatively slow under ambient conditions (half-life ~168 days) and is highly pH-dependent—degrading more rapidly in alkaline rather than acidic media [110,114,115]. Moreover, PLA’s low glass transition temperature (~55–60 °C) and slow crystallization kinetics limit its recyclability and post-use material recovery options [118].

The biological degradation of PHAs, in contrast, is more efficient and occurs under broader environmental conditions. Microbial synthesis from waste-derived feedstocks makes PHA production potentially sustainable and circular, although the high cost of its substrates and downstream processing remains a significant bottleneck [146,149]. PHA’s tunable properties—dictated by microbial strain, fermentation conditions, and carbon source—allow for diverse applications ranging from agricultural mulching films to medical implants [138,141,153].

From a material engineering perspective, crystallinity, molecular weight, and monomer sequence regularity are consistently shown to influence degradation kinetics [25,50,51]. Additionally, polymer matrices with high hydrophilicity and specific structural features (e.g., ester, keto, or ether groups) are more amenable to both abiotic hydrolysis and microbial enzymatic attack [72,73,253].

Another critical consideration is the end-of-life scenario. While biodegradability is attractive, it does not support monomer recovery like mechanical or chemical recycling [14]. Therefore, biodegradable polymers should be applied where degradation is an environmental necessity—such as in single-use food packaging, agricultural films, or biomedical devices that obviate secondary removal surgeries [141,284,290]. The notion that biodegradability alone solves the plastic problem is misleading unless matched with context-specific design, appropriate disposal infrastructure, and lifecycle assessments.

The excessive exploitation of non-renewable resources contributes significantly to greenhouse gas emissions and environmental pollution, thereby accelerating the degradation of the Earth’s ecosystems and climate. This has driven the need for renewable energy sources and alternative chemicals. Another pressing issue is the widespread use of petroleum-based plastics, which not only deplete global oil reserves but also result in plastic pollution due to inadequate waste disposal practices. This traditional “linear economy” assumes an abundance and the easy disposal of resources [499].

The persistent depletion of landfill space due to plastic waste necessitates the use of biodegradable polymers as alternatives to conventional, non-degradable plastics. These biodegradable and biocompatible polymers are now emerging as valuable substitutes for petroleum-based materials and can be produced from renewable raw materials [500]. Ryberg et al. reported that in 2015, approximately 6.2 million tons of macroplastics (>5 mm) and 3 million tons of microplastics (<3 mm) were lost to the environment out of the 322 million tons of plastic produced globally (excluding elastomers and synthetic fibers) [501].

A few words can also be said about the impact of biodegradable polymers on other sectors. For instance, the study in [502] explores the use of cross-linked polymer compositions as blocking agents during well-killing operations under conditions of high fracturing. Although not biodegradable in a strict environmental sense, such temporary polymeric systems highlight how polymer degradation and controlled breakdown can be engineered for specific subsurface applications, an approach conceptually aligned with the goals of environmentally responsive and biodegradable materials.

Furthermore, in [503], a novel application of the Hartmann–Sprenger effect for regulating natural gas pressure through energy separation mechanisms was introduced. This experimental research presents a non-thermal, quasi-isothermal pressure reduction system using nozzle–resonator pairs that converts pressure energy into heat without external energy input. While not directly addressing biodegradable polymers, this work underscores the broader potential of materials and system design to reduce energy consumption and environmental impact in industrial applications—a principle equally crucial in the development and deployment of biodegradable polymer systems.

Finally, the integration of bio-based content with biodegradability remains an ongoing challenge. Some polymers, like bioPET or PVA, may be bio-derived but not readily biodegradable without specific conditions [179,209]. Future development must balance renewable sourcing, ease of processing, functional performance, and environmental safety. Multidisciplinary collaboration, as noted in the Abstract and Introduction, is crucial to accelerate this balance and fulfill the potential of biodegradable polymers as sustainable material solutions [20,164,294,504].

## 8. Conclusions

Biodegradable polymers present a viable path toward reducing plastic pollution and promoting sustainable material cycles. While materials like PLA and PHAs have demonstrated significant potential, their adoption is constrained by technical, economic, and infrastructural barriers. Bridging these gaps requires a multidisciplinary approach that combines advances in polymer chemistry, microbial biotechnology, process engineering, and environmental science. Policymakers must also align certification standards with real-world degradation scenarios to ensure clarity and trust among end-users. Ultimately, the development and deployment of biodegradable polymers should be viewed not as a singular solution but as a crucial component within a broader strategy for sustainable material management.

## Figures and Tables

**Table 1:** Key points on PLA.

Category	Key Points	References
Chemical Nature and Synthesis	- PLA is a biodegradable thermoplastic made via the ring-opening polymerization (ROP) of lactide or azeotropic polycondensation of lactic acid.	[98,99,100,108,109,110]
	- First synthesized in 1932 by Wallace Carothers; commercialized in the late 1990s.	[111]
	- Lactic acid sourced from microbial fermentation of renewable carbohydrates.	[112,113,122,130]
	- Copolymerization with glycolide used to adjust degradation rates.	[101]
	- Catalysts: tin, aluminum, and zinc; novel catalysts include yttrium, zirconium, hafnium, gold, and platinum.	[100,120,121]
Material Properties	- Tg = 55–60 °C; exhibits slow crystallization and becomes tacky above Tg.	[118]
	- Mechanical properties (e.g., PLLA: tensile modulus ~3 GPa, tensile strength ~60 MPa, crystallinity ~37%).	[99]
	- High transparency and good processability.	[99,131,132,133]
Degradation Behavior	- Degrades abiotically by hydrolysis of ester bonds, followed by enzymatic breakdown (biotic phase).	[113,115,122]
	- Slower degradation in neutral/acidic pH; faster in alkaline conditions.	[114]
	- Base-catalyzed degradation involves transesterification and random ester bond cleavage.	[115]
Applications	- Packaging (films, containers, bottles, cups), biomedical (implants, sutures, drug delivery).	[102,103,104,105,106,107,123,124,125,126]
	- Textiles, hygienic products, tableware, mulch films, foamed PLA for cushioning/insulation.	[135,136,137]
	- Customizable for high-performance biomedical applications like scaffolds and tissue engineering.	[129]
Advantages	- Biodegradable, biocompatible, from renewable sources, processable by conventional plastic manufacturing.	[113,122,123,124,125,126]
	- Eco-friendly alternative to petroleum-based plastics; aligns with sustainability goals.	[122,130]
Limitations	- Slow degradation rate (~168 days half-life), low Tg, poor recyclability of some forms, higher production costs than conventional plastics.	[100,118]
	- Immiscibility of PLA with thermoplastic starch limits composite potential.	[118]

**Table 2:** Key points on PHAs.

Category	Key Points	References
Chemical Nature and Synthesis	- PHAs are aliphatic polyesters synthesized from β-, γ-, and δ-hydroxyalkanoic acids via the microbial fermentation of sugars and lipids.	[15,138]
	- Produced by microbial strains under nutrient stress (e.g., N, O, or P limitation) and carbon excess.	[139]
	- PHA properties depend on monomer side chain length and microbial strain used.	[147]
	- Accumulate as intracellular granules (0.2–0.7 µm) in bacteria.	[147]
Feedstocks and Sustainability	- Feedstocks include plant biomass, waste streams (municipal, industrial), wood chips, cardboard, and recycled plastics.	[148,149,150,151]
	- 30–50% of production cost comes from raw materials; waste-derived feedstocks reduce this.	[149]
	- Rice bran usable as feedstock; Sinorhizobium meliloti MTCC 100 is effective for biopolymer synthesis.	[152]
Closed-loop Production	- Waste converted to sugars → fermented to VFAs → used for PHA synthesis. - Pretreatment of waste can help, but may generate inhibitors (e.g., furfural). - Control of acidogenic inhibition and sustainable pretreatment are essential.	[146]
Material Properties	- Thermoplastic, biodegradable, and mechanically strong.	[138]
	- Properties adjustable by bacterial strain, substrate, and fermentation conditions.	[147]
Biodegradability and Eco-Friendliness	- Biodegradable, low energy requirements, non-toxic byproducts, minimal GHG emissions, support closed carbon cycle.	[148,149,153]
Applications	- Packaging (containers, films, bags), medical uses (sutures, scaffolds, wound dressings), agriculture (mulch films, seed coatings, fertilizers).	[16,138,141,142,143,145]
	- Used in printing toners, adhesives, textiles, toys, hygiene products, and electronics.	[144,154,160]
	- PHA-based packaging by companies like Biomers, P&G, Metabolix, etc. (e.g., shampoo bottles, surgical garments, upholstery).	[163]
Market Outlook	- Global PHA market projected at ~23,735 metric tons by 2021, with 6.27% CAGR.	[153]
Enhancement Techniques	- Performance enhanced by chemical modification or blending with other materials.	[155,156,157,158,159]

**Table 3:** Key points on other biodegradable polymers.

Polymer	Key Applications	Key Properties	Synthesis/Notes	References
PBS	Packaging, bags, mulch films	Low biocompatibility; improved in composites	Blended to enhance thermal/mechanical/gas/flame properties	[164,165,166]
PCL	Medical (sutures, scaffolds)	Biocompatible, slow degradation (1–2 years)	Ring-opening polymerization of caprolactone	[167,168,169,170,171,172]
PVA	Packaging, fibers, biomedical	Excellent film formation, thermal stability, water solubility	Biodegradable; crystallinity affects properties	[173,174,175,176,177,178,179,180,181,182,183]
PBAT	Compostable bags, wraps	Flexible, elongation (~700%), oil/water resistance	Polycondensation of adipic acid, terephthalic acid, and BDO	[184,185]
PEF/Furan Polyesters	Emerging bioplastics	No petrochemical counterpart	Made via polycondensation of 2,5-FDCA and glycol	[186,214]
PGA	Biomedical	Degrades via hydrolytic erosion	Linear aliphatic polyester	[187,188]
PLLA/PLA	Load-bearing, medical, packaging	Biodegradable, good mechanics, recyclable	High melting temp, high MW variants exist	[100,189,190,191,192,193,194,224,225,226,227,228,229,230]
PLGA	Biomedical (drug delivery)	Biodegradable, biocompatible	Copolymer of lactide and glycolide	[194,195]
PDLLA	Medical	Amorphous structure	Differs from crystalline PLLA	[196]
PTMC	Biomedical	Fully biodegradable	Ring-opening polymerization	[197]
Polyurethanes (PU)	Coatings, adhesives, foams	Properties depend on soft segments	Biodegradable with polyester polyols	[198,199,200,201,202,203,204]
Bio-PP	General-purpose plastic	Renewable source	Derived from bio-propylene	[205,206]
PPC	Impact-resistant plastics	Biodegradability enhanced via blends	Copolymer of propylene oxide + CO_2_	[208]
PET (bio/non-bio)	Packaging	Suggested for biodegradation, recyclable	Biodegradation via PETase enzyme possible	[209,210,235]
PDO	Biomedical	Fully biodegradable	Used in sutures and implants	[211,212,213]
Agropolymers/Biopolyesters	General bio-based plastic categories	PLA, PHA, etc.	Derived from renewable resources	[215]
Nonwoven Polymers (PLA, Bionolle)	Medical, hygiene, automotive	Breathable, compostable	Nonwovens from natural/bio-fibers	[216,217,218,219,220,221,222,223]
Natural Fibers (e.g., coir, kenaf)	Automotive, geotextiles, insulation	Good thermal/sound insulation	Used with recycled polymers	[219,220,221]
Bio-based PDO	Biopolymer production	High purity, economically viable	Microbial fermentation (DuPont process)	[231,232]
Bio-based Polyamides (BioPA)	Engineering plastics	High impact, abrasion resistance	Diacid + diamine or amino acid precursors	[233]
Bio-PET/Bio-PTT	Bottles, textiles	Partially bio-based	New methods to produce bio-based TA	[234]

**Table 4:** Biodegradable polymers in biomedical applications.

Polymer/Material	Key Properties	Biomedical Applications	Processing/Techniques	References
PGA (Polyglycolic Acid)	Strong, fast biodegradation	Sutures, orthopedic screws, bone treatment	Copolymerized with lactide (PGLA)	[236,237,238,239,240,241,242]
PLA (Polylactic Acid)	Biocompatible, thermoplastic, tailorable degradation	Sutures, implants, scaffolds, drug delivery, wound care, agricultural uses	Electrospinning, melt extrusion, gas foaming, TIPS, phase separation	[239,240,241,249,258,264,293,294,295,296,297,298,299,300,301,302,303]
PCL (Poly(ε-caprolactone))	Slow degradation, good flexibility	Drug delivery systems, implants, scaffolds	Copolymers with PLA, electrospinning	[249]
PDO (Polydioxanone)	Biodegradable, flexible	Medical devices	Approved in medical-grade formulations	[249]
PLGA (Poly(lactic-co-glycolic acid))	Variable degradation (days–years)	Controlled drug release, implants	Adjusting lactide–glycolide ratio	[250,251]
Chitin derivatives	High biodegradability	Wound healing, scaffold development	—	[200]
PHB (Poly(β-hydroxybutyrate))	Biodegradable, strong, biocompatible	Sutures, bone grafts, implants	Microbial synthesis from carbon-rich feedstocks	[252,288,289,290,291]
PHAs (Polyhydroxyalkanoates)	Renewable, biocompatible	Heart valves, vascular grafts, drug carriers	Industrial microbial production	[288,289,290,291]
PLA/O-MMT Nanocomposites	Enhanced strength, porosity, nanostructure	Scaffolds, foams, biomedical nanomaterials	Melt extrusion, CO_2_ foaming, selective extraction	[253,254,255,256,257,258]
PLA/Gelatin + EGF	Bioactive, nanostructured	Diabetic wound scaffolds	Electrospinning	[267]
Photo-crosslinked synthetic polymers	Tunable crosslinking/degradation	Drug delivery, cell encapsulation, tissue scaffolds	Additive manufacturing, stereolithography	[304,307]
Scaffold polymers (general)	Porous, bioresorbable	Tissue engineering, regenerative medicine	Salt leaching, freeze-drying, gas foaming, TIPS	[268,269,270,271,272,273,274,275,276,277,278,281,282,283]
Biodegradable nonwoven polymers	Micro/nanofibrous, agent-releasing	Wound/burn dressings	Electrospinning	[279,280]
Plasticized PHB	Improved flexibility, processability	Implants, packaging	Plasticizers (DBS, DOS, PEG, PIB), up to 20 wt%	[290]

**Table 5:** Summary of starch-based bioplastic development and properties.

Aspect	Details	References
Raw Material	Starch (from potato, rice, wheat, tapioca, corn, barley)	[400,401,402,414,416,429,430,431,432,433]
Key Advantages	- Biodegradable; - Renewable; - Abundant; - Low-cost.	[400,401,402,409,413,423]
Main Development Strategies	Blending starch with polyolefins (e.g., PE, PP); Thermoplastic starch (TPS) + biodegradable polymers.	[402,403,404,405,406,425]
Environmental Impact	Reduces reliance on petroleum resources; decomposes naturally	[407,410,411,412,439]
Thermoplastic Conversion	Requires plasticizers (e.g., glycerol, water, urea); processed under heat and shear	[419,420,441]
Additives Used	Natural fillers, essential oils, nanoparticles, PLA, BHET, PVA	[419,424]
Functional Enhancements	Increased tensile strength, flexibility, barrier properties	[422,423,438]
Common Applications	Biowaste bags, trays, mulch films, plant pots, cosmetics, food packaging	[417,418,425]
Commercial Products	Mater-Bi by Novamont S.p.A.	[428]
Processing Techniques	Casting (lab-scale), thermal processing (preferred for scale-up), extrusion	[435,436,437,438]
Film Characteristics	Transparent, odorless, tasteless, good mechanical and barrier properties	[435,436,437]
Challenges	- Not inherently thermoplastic; - Retrogradation; - Hydrophilicity; - Limited casting scalability.	[419,438]
Solutions for Challenges	- Plasticizers; - Surface modification (e.g., silane); - Use of pro-oxidants; - Use of lipids and hydrocolloids.	[420,421,438]
Mechanical Performance	E.g., TPS/PLA/CNF nanocomposites: ~37 MPa tensile strength, ~630 MPa Young’s modulus	[422]
Nanofillers	Nanocellulose, nanoclays, metal oxides	[423,424]
Physicochemical Influences	- Amylose: tensile strength ↑, elongation ↓; - Amylopectin: crystallinity ↑.	[409]
Modification Methods	Chemical derivatization, cross-linking, enzymatic saccharification, ultrasonication	[415,440,442]
Recent Advances	Tapioca starch + sugarcane bagasse fiber composites; improved via ultrasonication	[442]
Market Share (2021)	Starch-based blends ≈ 16.4% of global bioplastic production	[424]
Starch’s Role in Biopolymer Production	Fermented to glucose → lactic acid → PLA	[434]
